# Systematic and biogeographical study of Protura (Hexapoda) in Russian Far East: new data on high endemism of the group

**DOI:** 10.3897/zookeys.424.7388

**Published:** 2014-07-08

**Authors:** Yun Bu, Mikhail B. Potapov, Wen Ying Yin

**Affiliations:** 1 Institute of Plant Physiology and Ecology, Shanghai Institutes for Biological Sciences, Chinese Academy of Sciences, Shanghai, 200032 China; 2 Moscow State Pedagogical University, Kibalchich str., 6, korp. 5, Moscow, 129278 Russia

**Keywords:** Key, checklist, *Baculentulus*, *Fjellbergella*, *Yichunentulus*, biogeography

## Abstract

Proturan collections from Magadan Oblast, Khabarovsk Krai, Primorsky Krai, and Sakhalin Oblast are reported here. Twenty-five species are found of which 13 species are new records for Russian Far East which enrich the knowledge of Protura known for this area. Three new species *Baculentulus krabbensis* **sp. n.**, *Fjellbergella lazovskiensis* **sp. n.** and *Yichunentulus alpatovi* **sp. n.** are illustrated and described. The new materials of *Imadateiella sharovi* (Martynova, 1977) are studied and described in details. Two new combinations, *Yichunentulus borealis* (Nakamura, 2004), **comb. n.** and *Fjellbergella jilinensis* (Wu & Yin, 2007), **comb. n.** are proposed as a result of morphological examination. Keys to species of the genera *Fjellbergella* and *Yichunentulus* are given. An annotated list of all species of Protura from Russian Far East is provided and discussed. Widely distributed species were not recorded in this area. This may be because of the high sensitivity of Protura to anthropogenic impact and low dispersal ability of the group.

## Introduction

The Protura are minute soil-dwelling arthropods with more than 800 species known so far in the world (Bu et al. 2012, Szeptycki 2007, Shrubovych 2014). They have been a group of focus in the study of the evolutionary history of Hexapoda and Arthropoda because of their basal phylogenetic position (Chen et al. 2011, Luan et al. 2005, Mallatt et al. 2010, Meusemann et al. 2010).

**Figure 1.**
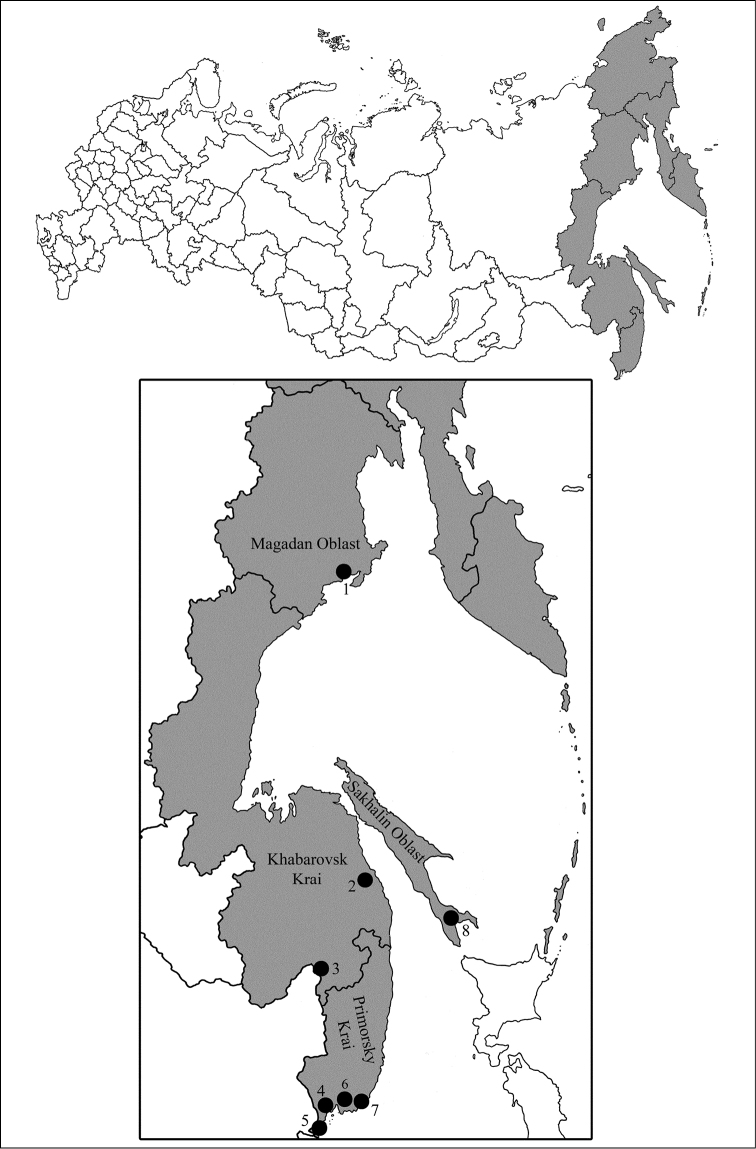
The sampling sites in RFE.

The Russian Far East (abbreviated as RFE throughout the present paper) occupies an area of 3,016 thousand sq. km. and extends from Wrangel Island southwards to Khasan Lake (Fig. 1). Forests occupy 39% of the territory and predominate in Primorsky Krai, Amur Oblast, Sakhalin Oblast, and Khabarovsk Lrai. The insect fauna is associated with mixed broadleaved-coniferous forests in the south of RFE. About 31,500 species of insects have been recorded in the RFE so far where Eastern-Asiatic and local species make the most part of the fauna (Storozhenko et al. 2002). Eighteen species of Protura have been recorded in the RFE. Compared to the neighbouring regions, 34 species of Protura have been recorded in Northeast China (Bu et al. 2013), 20 species were found in Korea (Lee and Rim 1988) and 88 species are recorded in Japan (Kaneko et al. 2012). The only seven publications on Protura of the RFE are: Martynova who described *Imadateiella sharovi* (Martynova, 1977) from Magadan; Nakamura (2004) who reported eight species from Khabarovsk Krai; Shrubovych (2009, 2010, 2014) and Shrubovych and Bernard (2012, 2013) who studied the Protura materials from different collections of Primorsky Krai and Sakhalin Island and nine species have been added to the proturan fauna of RFE.

Much of the material was collected by us during a joint Chinese-Russian expedition in autumn of 2011 which was a part of study of the biodiversity of basal Hexapoda of Pacific coast of Asia (2011–2012). Several localities in southern RFE were searched: three locations of Primorsky Krai (Shkotovsky, Khasansky, and Lazovsky districts) and one of Khabarovsk Krai (Vaninsky district). Other materials collected by M. Potapov et al. (2009–2010) were also used. Around a thousand individuals are included in our study. Based on this material in all 25 species were identified, including 3 new species and 10 species newly recorded for the RFE. For another 12 species we give the new data on their distribution. The genera *Fjellbergella* and *Yichunentulus* are revised and rediagnosed, and the keys to species of the two genera are given.

## Materials and methods

The specimens were collected by Tullgren funnels using fast extraction with strong heating of samples during several hours. All specimens were mounted on slides in Hoyer’s medium and dried at 60 °C. Specimens were identified and drawn with the aid of a NIKON E600 phase contrast microscope. The photos were taken by digital camera Nikon DXM1200. Type specimens are deposited in the Shanghai Entomological Museum (SEM), Institute of Plant Physiology & Ecology, Shanghai Institutes for Biological Sciences, Chinese Academy of Sciences, and Moscow State Pedagogical University (MSPU).

Eight localities of RFE were sampled so far (Fig. 1) if including our collecting (denoted as 2–5 and 7); localities 1, 6 and 8 were studied in previous publications:

1. Magadan Oblast, Snezhnaja dolina, 59°32.92'N, 150°48.16'E.
2. Khabarovsk Krai, Vaninsky area, Valley of Mulinka, 49°49.42'N, 140°0.50'E.
3. Khabarovsk Krai, Khabarovsk district, Korfovsky, Khekhtsyr Range, 48°30.95'N, 135°6.06'E.
4. Primorsky Krai, Shkotovsky area, Anisimovka, Khualaza Mt. 43°10.33'N, 132°47.16'E.
5. Primorsky Krai, Khasansky area, 42°37.40'N, 130°52.35'E.
6. Primorsky Krai, Partizansky area, Chondolaz Range, 42°45.97'N, 133°3.75'E.
7. Primorsky Krai, Lazovsky area, nearby Preobrazheniye, 42°54.16'N, 133°53.33'E.
8. Sakhalin Oblast, Sakhalin Island, Yuzhno-Sakhalinsk, Susunaysky Range, Chenov Mt. 47°1.50'N, 142°52.35'E.

Abbreviations used in the text follow the paper of Bu and Yin (2007). Head setae and pores are marked according to Rusek et al. (2012). Arrangements of the taxa follow the system proposed by Yin (1999).

## Results

### Systematics

#### Descriptions of new species

##### Baculentulus krabbensis sp. n.

Taxon classificationAnimaliaProturaBerberentulidae

http://zoobank.org/2AD017F3-F73F-4AF5-A0FE-C317387B9A77

**Figure 2.**
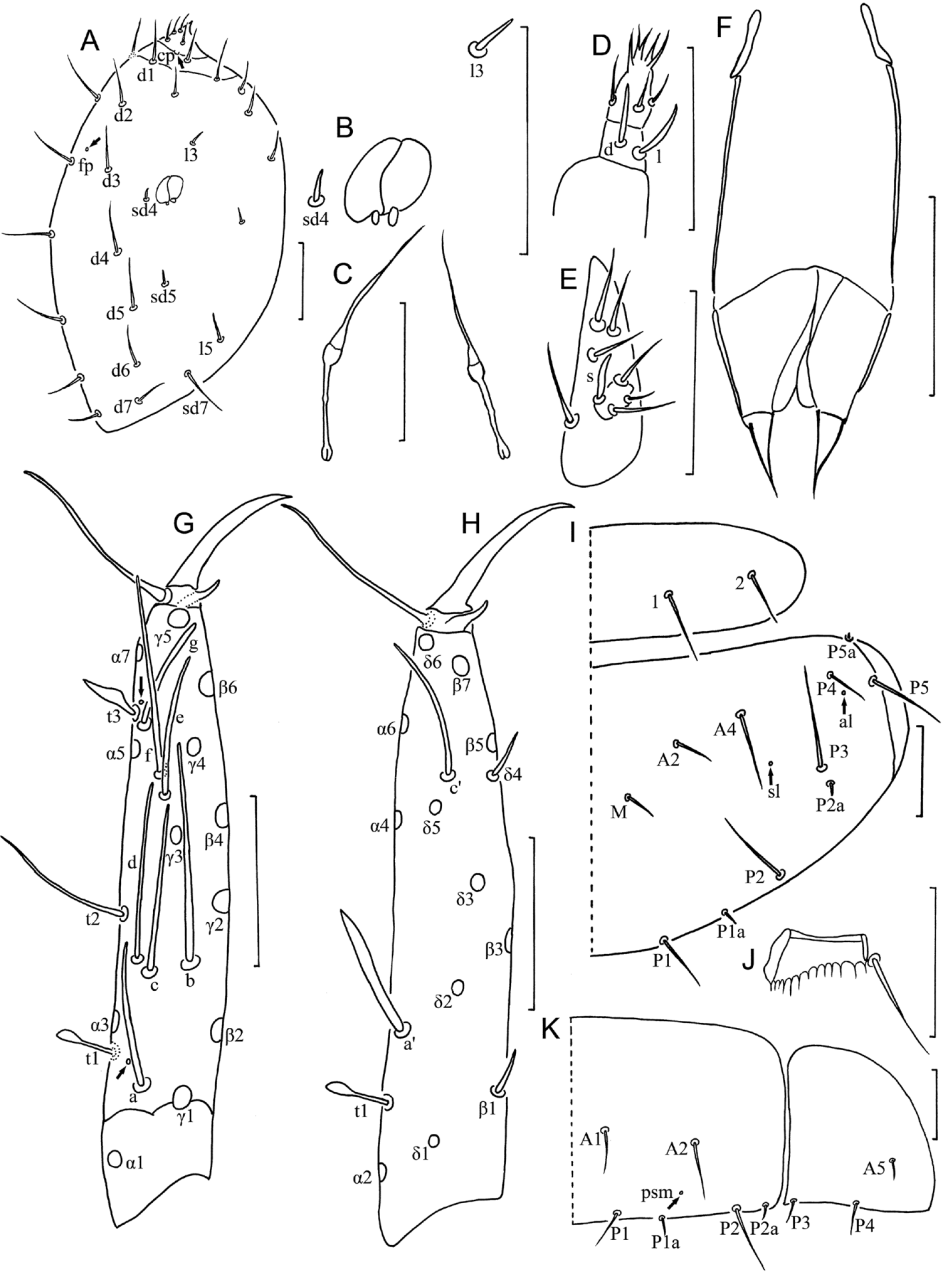
*Baculentulus krabbensis* sp. n. holotype. **A** Head, dorsal view (*cp* = clypeal pore, *fp* = frontal pore) **B** pseudoculus **C** canal of maxillary gland **D** maxillary palpus **E** labial palpus **F** female quama genitalis **G** foretarsus, exterior view **H** foretarsus, interior view **I** pronotum and mesonotum, right side (*al* = anterolateral pore, *sl* = sublateral pore) **J** comb **K** tergite I, right side. Arrows indicate pores. Scale bar: 20 μm.

**Figure 3.**
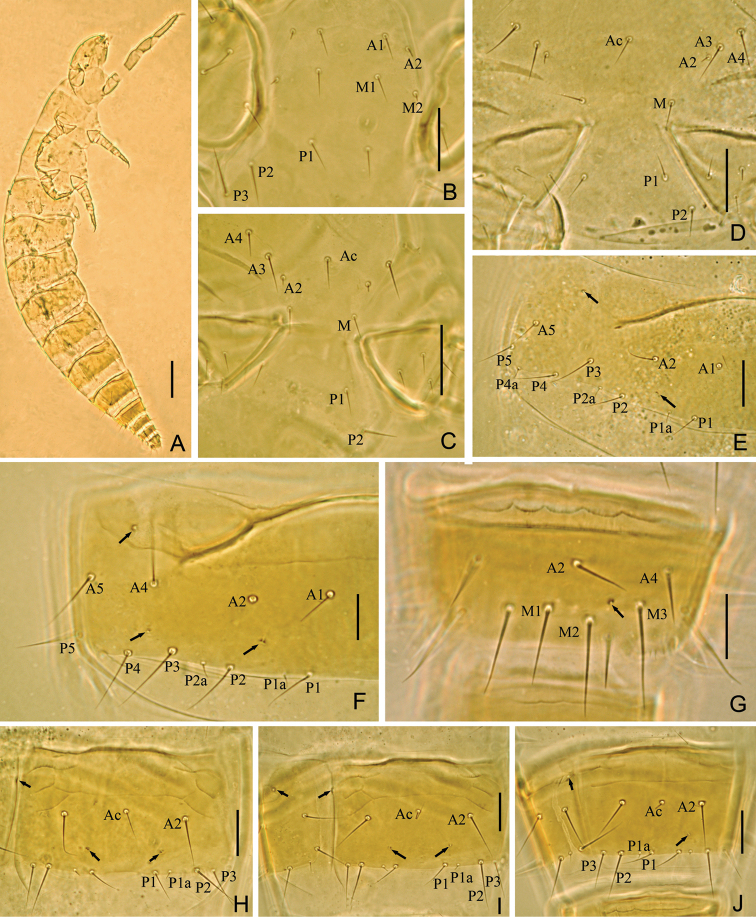
*Baculentulus krabbensis* sp. n. holotype. **A** Habitus **B** prosternum **C** mesosternum **D** metasternum **E** tergite III, left side **F** tergite VI, left side **G** tergite VIII **H**–**J** sternites V–VII. Arrows indicate pores. Scale bar: 100 μm in **A**, others, 20 μm.

Figs 2 , 3 ; Table 1

###### Material examined.

Holotype, female (No. FE-2011022-2) (SEM), Russia, Far East, Primorsky Krai, Khasansky area, eastern part of Krabbe Peninsula, from mixed samples of soil and moss under a broad-leaved forest on a hill beside the coast, 42°37.40'N, 130°52.35'E, 16-IX-2011, coll. Y. Bu, C. W. Huang, M. Potapov & V. Alpatov. Paratypes, 2 females (Nos. FE-2011022-1, FE-2011023) (SEM; MSPU), 1 maturus junior (No. FE-2011022-3) (SEM), same data as holotype.

###### Description.

Adult body length 1000–1100 µm (n=3), pale yellow in color (Fig. 3A).

**Head** (Fig. 2A). Ovate, length 90–110 µm, width 70 µm. Setae *d6* present, *sd4* and *sd5* short sensilliform. Setae *d6* 11 µm, *d7* 10 µm and *sd7* 19 µm in length. Clypeal pore *cp* and frontal pore *fp* present. Pseudoculus round, length 7–8 µm, with short posterior extension, PR=15 (Fig. 2B). Calyx of maxillary gland smooth, without any appendix, blind end split into two leaves, posterior filament 15 µm, CF=7 (Fig. 2C). Maxillary palpus with two tapering sensilla, subequal in length (Fig. 2D). Labial palpus reduced, with three setae and one lanceolate basal sensillum (Fig. 2E).

**Foretarsus** (Fig. 2G, H). Length 80 µm, claw length 20 µm, without inner flap, TR=4; empodium length 3 µm, EU=0.15. Dorsal sensilla *t-1* baculiform, *t-2* slender and long (17 µm), BS=0.33, *t-3* broad-leaf shape. All other exterior sensilla slender, except broadened sensillum *g*, *a* surpassing base of *b* and *c*, *b* broad at base and extremely long (29 µm), reaching base of *γ4*, *c* nearly reaching base of *e* and slightly lower than *b* and *d*, *d* located at same level to *b*, *e* short, *f* long, *g* broad and short. Interior sensilla *a*’ lanceolate and broad, *b*’ absent, *c*’ reaching base of claw. Relative length of sensilla: *t1* = *t3* < *a*’ < (*g* = *t2 = c*’) < *a* < (*c* = *e*) < *d* < *f* < *b*. Setae *β1* and *δ4* sensillum, 6 µm and 7 µm respectively. Pores close to base of sensilla *a* and *t3* present. Length of middle tarsus 35 µm, claw length 15 µm. Length of hind tarsus 38 µm, claw length 18 µm.

**Thorax.** Thoracic chaetotaxy given in Table 1. Setae *1* and *2* on pronotum 16 µm and 12 µm length respectively, mesonotum and metanotum with eight pairs of *P*-setae, accessory setae short sensilliform; setae *P1*, *P1a* and *P2* on mesonotum 15 µm, 3 µm and 20 µm respectively (Fig. 2I). Prosternum with two pairs of anterior seta, and setae *A2* and *M2* sensilliform (Fig. 3B). Mesosternum and metasternum each with 7 *A*-setae, and setae *A2* sensilliform (Fig. 3C, D). Pronotum and prosternum without pores (Figs 2I, 3B). Mesonotum with pores *sl* and *al*, metanotum with pores *sl* only (Fig. 2I). Mesosternum and metasternum without pores (Fig. 3C, D). Single membranal pore present on membrane between each coxa and the body.

**Table 1.** Adult chaetotaxy of *Baculentulus krabbensis* sp. n.

Segment		Dorsal		Ventral	
		Formula	Setae	Formula	Setae
Th.	I	4	1, 2	(4+4)/6	A1, 2, M1, 2 P1, 2, 3
	II–III	6/16	A2, 4, M P1, 1a, 2, 2a, 3, 4, 5, 5a	(7+2)/4	Ac, 2, 3, 4, M Pc, 1, 2
Abd.	I	6/12	A1, 2, 5 P1, 1a, 2, 2a, 3, 4	3/4	Ac, 2 P1, 2
	II–III	6/16	A1, 2, 5 P1, 1a, 2, 2a, 3, 4, 4a, 5	3/5	Ac, 2 Pc, 1a, 2
	IV–V	6/16	A1, 2, 5 P1, 1a, 2, 2a, 3, 4, 4a, 5	3/8	Ac, 2 P1, 1a, 2, 3
	VI	8/16	A1, 2, 4, 5 P1, 1a, 2, 2a, 3, 4, 4a, 5	3/8	Ac, 2 P1, 1a, 2, 3
	VII	8/18	A1, 2, 4, 5 P1, 1a, 2, 2a, 3, 3a, 4, 4a, 5	3/8	Ac, 2 P1, 1a, 2, 3
	VIII	6/16	A2, 4, 5 M1, 2, 3, 4, P2, 3, 4, 5	4	A1, 2
	IX	14	1, 1a, 2, 2a, 3, 3a, 4	4	1, 2
	X	12	1, 1a, 2, 2a, 3, 4	4	1, 2
	XI	6	1, 3, 4	6	1, 1a, 2
	XII	9		6	

**Abdomen.** Abdominal chaetotaxy given in Table 1. Tergite I with three pairs of anterior setae (*A1*, *A2*, A5) and six pairs of posterior setae (Fig. 2K). Tergites II–VI with eight pairs of posterior setae. Tergite VI and VII with four pairs of anterior setae (*A1*, *A2*, *A4*, *A5*). Tergites VII with nine pairs of posterior setae, *P3a* present. All accessory setae on tergites I–VII sensilliform, 5–6 µm on tergites I–VI and 7 µm on VII. Tergite VIII with paired setae *M1* (Fig. 3G). Sternite IV–VII each with eight posterior setae, *Pc* absent (Fig. 3H, I, J). Sternite VIII with 4 setae.

Tergites I and VIII with pores *psm* only (Figs 2K, 3G), II–V with pores *psm* and *al* (Fig. 3E), VI–VII with pores *psm*, *al* and *psl* (Fig. 3F), IX–XI without pores, XII with single medial pore. Sternites I–III without pores, IV with 1+1 anteromembranal pores, V and VI each with 1+1 posterior pores anterior to seta *P1* and 1+1 anteromembranal pores (Fig. 3H, I), VII with single posterior pore asymmetrical located left or right and 1+1 anteromembranal pores (Fig. 3J), VIII–XI without pores, XII with 1+1 pores *al*.

Abdominal appendages I, II, III with 2, 1, 1 segments and 4, 2, 2 setae respectively. On appendages II and III, subapical seta 15–17 µm, apical seta 11–12 µm in length. Striate band on abdominal segment VIII reduced, anterior margin regular wave shaped (Fig. 3G). Comb on abdomen VIII rectangular, with 12–13 teeth (Fig. 2J). Female squama genitalis with very short basal apodeme, extremely long and pointed acrostyli (Fig. 2F). Male unknown.

###### Etymology.

The species is named after Krabbe Peninsula where the type specimens were collected.

###### Distribution.

Known only from type locality.

###### Diagnosis.

*Baculentulus krabbensis* sp. n. is characterized by extremely long sensilum *b* on foretarsus, sensillum *a*’ located distal to *t1*, sensillum *b*’ absent, eight *A*-setae on tergite VI and VII, presence of *P3a* on tergite VII, and special female genitalia with short basal apodeme.

###### Remarks.

We placed the present new species in the genus *Baculentulus* because the baculiform sensillum *t1* on foretarsus, reduced labial palpus with three setae and one sensillum, smooth calyx of maxillary gland, reduced striate band, two pairs of anterior setae on mesonotum and metanotum, abdominal appendages II and III each with two setae of different length, and only 4 setae on sternite VIII. It is similar to *Baculentulus samchonri* (Imadaté & Szeptycki, 1976) from North Korea in having eight *A*-setae on both tergites VI and VII, absence of sensillum *b*’ and extremely long sensillum *b* on foretarsus. They can be distinguished by the posterior setae on tergite VII (9 pairs of *P*-setae with *P3a* present in *Baculentulus krabbensis* sp. n. vs. 8 pairs of *P*-setae with *P3a* absent in *Baculentulus samchonri*), the anterior setae on tergite I (6 A-setae in *Baculentulus krabbensis* sp. n. vs. 4 in *Baculentulus samchonri*), the length of sensillum *f* (extremely long and surpassing the base of claw in *Baculentulus krabbensis* sp. n. vs. short and not reaching base of claw in *Baculentulus samchonri*), and the shape of female squama genitalis (basal apodeme very short in *Baculentulus krabbensis* sp. n. vs. basal apodeme in moderate length in *Baculentulus samchonri*).

##### Fjellbergella lazovskiensis sp. n.

Taxon classificationAnimaliaProturaAcerentomidae

http://zoobank.org/152D05AF-CE80-4BDA-B821-833163392DC1

**Figure 4.**
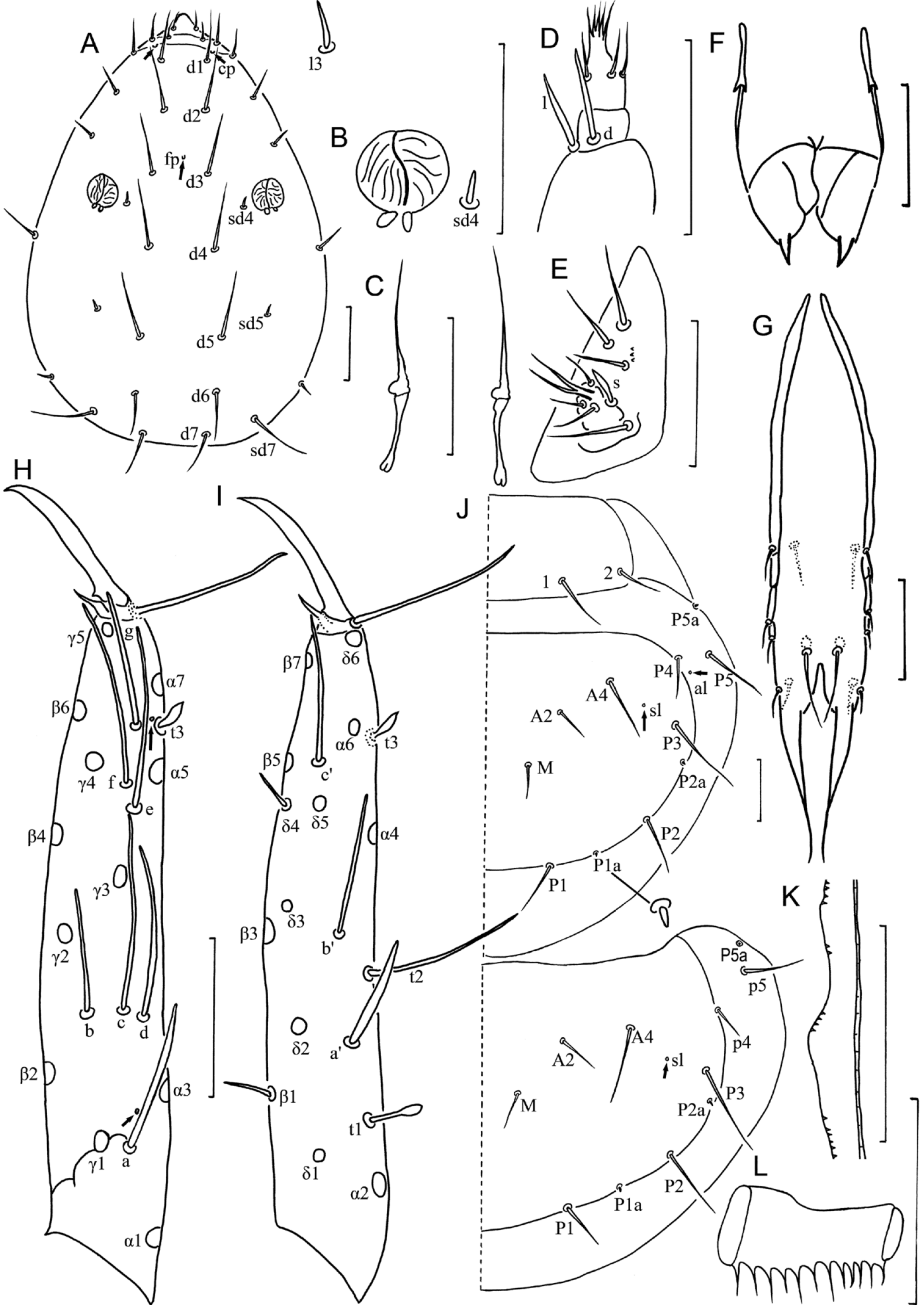
*Fjellbergella lazovskiensis* sp. n. holotype. **A** Head, dorsal view **B** pseudoculus **C** canal of maxillary gland **D** maxillary palpus **E** labial palpus **F** female squama genitalis **G** male squama genitalis **H** foretarsus, exterior view **I** foretarsus, interior view **J** nota, right side **K** part of striate band **L** comb. Arrows indicate pores. Scale bar: 20 μm.

**Figure 5.**
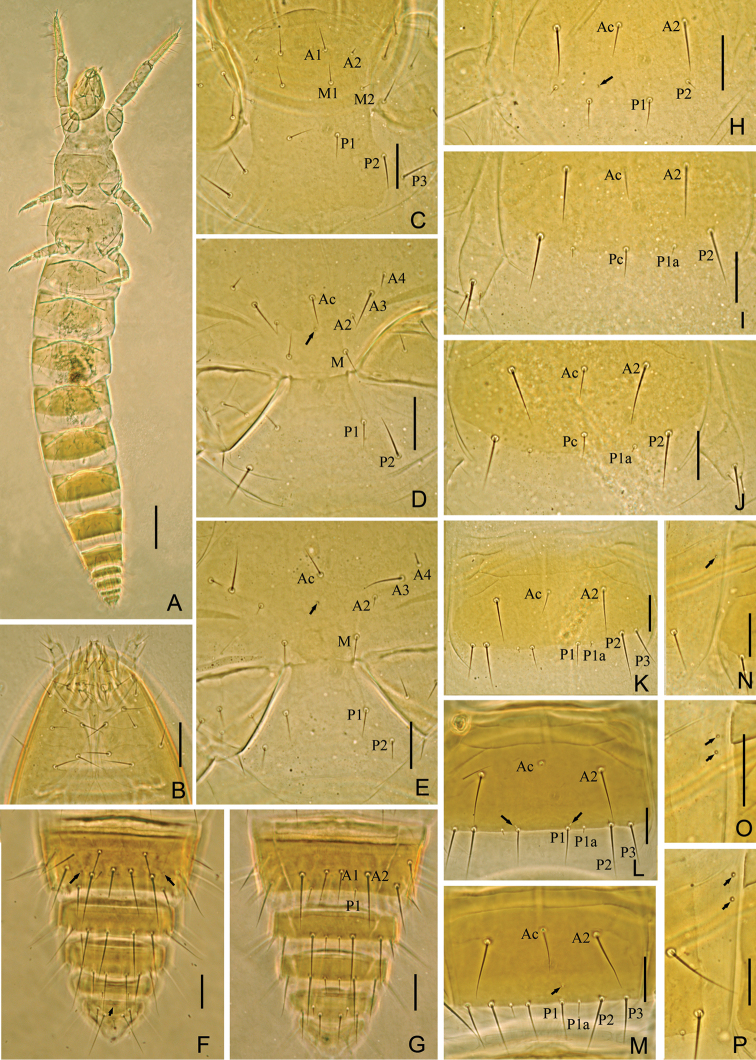
*Fjellbergella lazovskiensis* sp. n. holotype. **A** Habitus **B** ventral side of head **C** prosternum **D** mesosternum **E** metasternum **F** tergites VIII–XII **G** sternites VIII–XII **H–J** sternites I–III **K** sternite IV **L** sternite VI **M** sternite VII **N–P** laterotergites of abdominal segments IV, V and VI. Arrows indicate pores. Scale bar: 100 μm in **A**, others, 20 μm.

Figs 4 , 5 ; Table 2

###### Material examined.

Holotype, female (No. FE-2011051-2) (SEM), Russia, Far East, Primorsky Krai, Lazovsky area, nearby Preobrazheniye, from the mixed samples of humus (full of white mycelium) and a little soil under a broad-leaved forest close to the seashore, 42°54.16'N, 133°53.33'E, 22-IX-2011. coll. Y. Bu, C. W. Huang, M. Potapov & V. Alpatov. Paratypes, 5 females (Nos. FE-2011046-2, FE-2011052-4, FE-2011052-5, FE-2011052-6, FE-2011073-1) (SEM), 5 males (Nos. FE-2011071-1, FE-2011072-1, FE-2011074-1, FE-2011075-1, FE-211075-3) (SEM; MSPU), same data as holotype. Other materials, 3 male preimagos (Nos. FE-2011048-2, FE-2011048-3, FE-2011049-3) (SEM), same date as holotype.

###### Description.

Adult body length 1100–1300 µm (n=10), pale yellow in color (Fig. 5A).

**Head** (Fig. 4A). Ovate, length 120–125 µm, width 85 µm. Setae *d6* present, *sd4* and *sd5* short sensilliform. Setae *d6* and *d7* subequal in length. Seta *sd7* 18 µm in length. Clypeal pore *cp* and frontal pore *fp* present. Pseudoculus round, length 9–10 µm, with short posterior extension, some irregular lines visible under light microscope, PR=12–13 (Fig. 4B). Calyx of maxillary gland smooth, with one helmet-like dorsal appendix, blind end split into two leaves, and posterior filament 15 µm, CF=8 (Fig. 4C). Maxillary palpus with two tapering sensilla, dorsal one (8–10 µm) slightly longer than lateral one (7–9 µm) (Fig. 4D). Labial palpus reduced, with two-branched terminal tuft of setae, with one leaf-shape basal sensillum (Figs 4E, 5B).

**Foretarsus** (Fig. 4H, I). Length 90–95 µm, claw length 20–21 µm, without inner flap, TR=4.3–4.7; empodium length 4 µm, EU=0.2. Dorsal sensilla *t-1* claviform, *t-2* slender and long (25–26 µm), BS=0.38–0.42, *t-3* short and lanceolate. All other exterior sensilla slender, with except *a* slightly broad, surpassing base of *c*, *b* shorter than *c* and located in subequal level, *d* lower than *c*, *e* slender, *f* long, *g* short. Interior sensilla *a*’ short and broad, *b*’ slender and surpassing base of *δ5*, *c*’ reaching base of claw. Relative length of sensilla: *t3* < *t1*< *a*’ < *b* < (*a* = *g* = *d* = *b*’ = *c*’) < (*c* = *e* = *f* = *t2*). Setae *β1* and *δ4* sensillum, 7 µm and 5 µm respectively. Pores close to base of sensilla *a* and *t3* present. Length of middle tarsus 40–45 µm, claw length 18–20 µm. Length of hind tarsus 45–50 µm, claw length 20–23 µm.

**Thorax.** Thoracic chaetotaxy given in Table 2. Setae *1* and *2* on pronotum 20 µm and 15 µm length respectively, mesonotum and metanotum with eight pairs of *P*-setae, accessory setae short sensilliform, 2.5 µm; setae *P1*, *P1a* and *P2* on mesonotum 18–20 µm, 2.5 µm and 22–25 µm respectively (Fig. 4J). Prosternum with two pairs of anterior seta, and setae *A2* and *M2* sensilliform (Fig. 5C). Mesosternum and metasternum each with 7 *A*-setae, and setae *A2* and *A4* sensilliform (Fig. 5D, E). Pronotum and prosternum without pores (Figs 4J, 5C). Mesonotum with pores *sl* and *al*, metanotum with pores *sl* only (Fig. 4J). Mesosternum and metasternum each with single median pore, situated anterioral to level of setae *M* (Fig. 5D, E).

**Table 2.** Adult chaetotaxy of *Fjellbergella lazovskiensis* sp. n.

Segment		Dorsal		Ventral	
		Formula	Setae	Formula	Setae
Th.	I	4	1, 2	(4+4)/6	A1, 2, M1, 2 P1, 2, 3
	II–III	6/16	A2, 4, M P1, 1a, 2, 2a, 3, 4, 5, 5a	(7+2)/4	Ac, 2, 3, 4, M Pc, 1, 2
Abd.	I	6/12	A1, 2, 5 P1, 1a, 2, 2a, 3, 4	3/4	Ac, 2 P1, 2
	II–III	6/16	A1, 2, 5 P1, 1a, 2, 2a, 3, 4, 4a, 5	3/5	Ac, 2 Pc, 1a, 2
	IV–V	6/16	A1, 2, 5 P1, 1a, 2, 2a, 3, 4, 4a, 5	3/8	Ac, 2 P1, 1a, 2, 3
	VI	8/16	A1, 2, 4, 5 P1, 1a, 2, 2a, 3, 4, 4a, 5	3/8	Ac, 2 P1, 1a, 2, 3
	VII	8/18	A1, 2, 4, 5 P1, 1a, 2, 2a, 3, 3a, 4, 4a, 5	3/8	Ac, 2 P1, 1a, 2, 3
	VIII	6/16	A2, 4, 5 M1, 2, 3, 4, P2, 3, 4, 5	4/2	A1, 2 P1
	IX	14	1, 1a, 2, 2a, 3, 3a, 4	4	1, 2
	X	12	1, 1a, 2, 2a, 3, 4	4	1, 2
	XI	6	1, 3, 4	6	1, 1a, 2
	XII	9		6	

**Abdomen.** Abdominal chaetotaxy given in Table 2. Tergite I with three pairs of anterior setae (*A1*, *A2*, A5) and six pairs of posterior setae. Tergites II–VI with eight pairs of posterior setae. Tergite VI and VII with four pairs of anterior setae (*A1*, *A2*, *A4*, *A5*). Tergite VII with nine pairs of posterior setae, *P3a* present. All accessory setae on tergites I–VII short sensilliform, 3 µm on tergites I–VI and 6 µm on VII. Tergite VIII with paired setae *M1* (Fig. 5F). Sternites IV–VII each with eight posterior setae, *Pc* absent (Fig. 5K, L, M). Sternite VIII with two rows of setae (4/2) (Fig. 5G).

Tergites I and VIII with pores *psm* only, II–VII with pores *psm* and *al*, IX–XI without pores, XII with single medial pore (Fig. 5F). Sternites I–III without pores (Fig. 5H–J), IV with 1+1 anteromembranal pores (Fig. 5K, N), V and VI each with 1+1 posterior pores close to seta *P1* and 2+2 anteromembranal pores (Fig. 5L, O, P), VII with single posterior pore asymmetrical located left or right (Fig. 5M), VIII–XI without pores, XII with 1+1 pores *al*.

Abdominal appendages I, II, III with 2, 1, 1 segments and 4, 3, 3 setae respectively. On appendages II and III, subapical seta 19–21 µm, two apical setae 11–12 µm, and 5–7 µm in length (Fig. 5I–J). Striate band on abdominal segment VIII reduced, anterior margin with sparse irregular teeth (Figs 4K, 5F, G). Comb on abdomen VIII rectangular, with 12 teeth (Fig. 4L). Female squama genitalis with short subuliform acrostyli (Fig. 4F). Male squama genitalis with 5+5 setae on dorsal side and 3+3 setae on ventral side (Fig. 4G).

**Chaetal variability.** Chaetal variations were observed in 4 specimens: on tergite VI, asymmetrical absence of *A4* of right side (No. FE-2011071-1); on tergite VII, asymmetrical absence of *P1* of right side (No. FE-2011052-4); on sternite VII, absence of *Ac* (No. FE-2011075-1), and asymmetrical absence of *A2* of left side (No. FE-2011052-6).

###### Etymology.

The species is named after Lazovsky Nature Reserve where the type specimens were collected.

###### Distribution.

Known only from type locality.

###### Diagnosis.

*Fjellbergella lazovskiensis* sp. n. is characterized by three pairs of anterior setae on tergites II-V which is different to any other members of the genus, four pair of anterior setae, nine pairs of posterior setae on tergite VII, foretarsal sensilla *b*, *c* and *d* located in subequal level, *b* shorter than *c*, and tergite IX and X with 14, 12 setae respectively.

###### Remarks.

The present species is located in the genus *Fjellbergella* because three setae on abdominal legs, labial palpus with two-branched terminal tuft of setae, claviform sensillum *t1* on foretarsus, reduced striate band, two pairs of anterior setae on mesonotum and metanotum, and 4/2 setae on sternite VIII. *Fjellbergella lazovskiensis* sp. n. is similar to *Fjellbergella tuxeni* Nosek, 1980 from Alaska in having 8 *A*-setae on tergite VII and presence of *P1a* on tergite I–VII. They can be distinguished by the chaetotaxy of tergite IX and X (14 and 12 setae in *Fjellbergella lazovskiensis* sp. n. respectively vs. 12 and 8 setae in *Fjellbergella tuxeni*), length and location of foretarsal sensilla *b* and *c* (*b* shorter than *c* and they located at the same level in *Fjellbergella lazovskiensis* sp. n. vs. *b* and *c* subequal in length and *c* located lower than *b* distinctly in *Fjellbergella tuxeni*), shape and length of sensillum *a*’ (broad and reaching base of sensillum *b*’ in *Fjellbergella lazovskiensis* sp. n. vs. slender and far surpassing base of sensillum *b*’ in *Fjellbergella tuxeni*), and shape of claw of foreleg (absence of inner flap in *Fjellbergella lazovskiensis* sp. n. vs. presence in *Fjellbergella tuxeni*). In addition, the new species has only 6 anterior setae on tergites II-V contrary to two other members of this genus, which have 8 anterior setae.

##### Yichunentulus alpatovi sp. n.

Taxon classificationAnimaliaProturaAcerentomidae

http://zoobank.org/513B6344-D78B-4777-90A9-AEB3BAEAB83E

Fig. 6 ; Table 3

###### Material examined.

Holotype, female (No. FE-2011035-1) (SEM), Russia, Far East, Primorsky Krai, Lazovsky area, nearby Preobrazheniye, from the mixed samples of soil and humus from mountains, 42°54. 48'N, 133°53. 96'E, 21-IX-2011, coll. Y. Bu, C. W. Huang, M. Potapov & V. Alpatov. Paratypes, 1 female (No. FE-2011035-6), 3 males (Nos. FE-2011035-4, FE-2011037-1, FE-2011045-3) (SEM; MSPU), 2 male preimagos (Nos. FE-2011038-1, FE-2011038-2) (SEM), same data as holotype.

###### Description.

Adult body length 1200–1350 µm (n=5).

**Figure 6.**
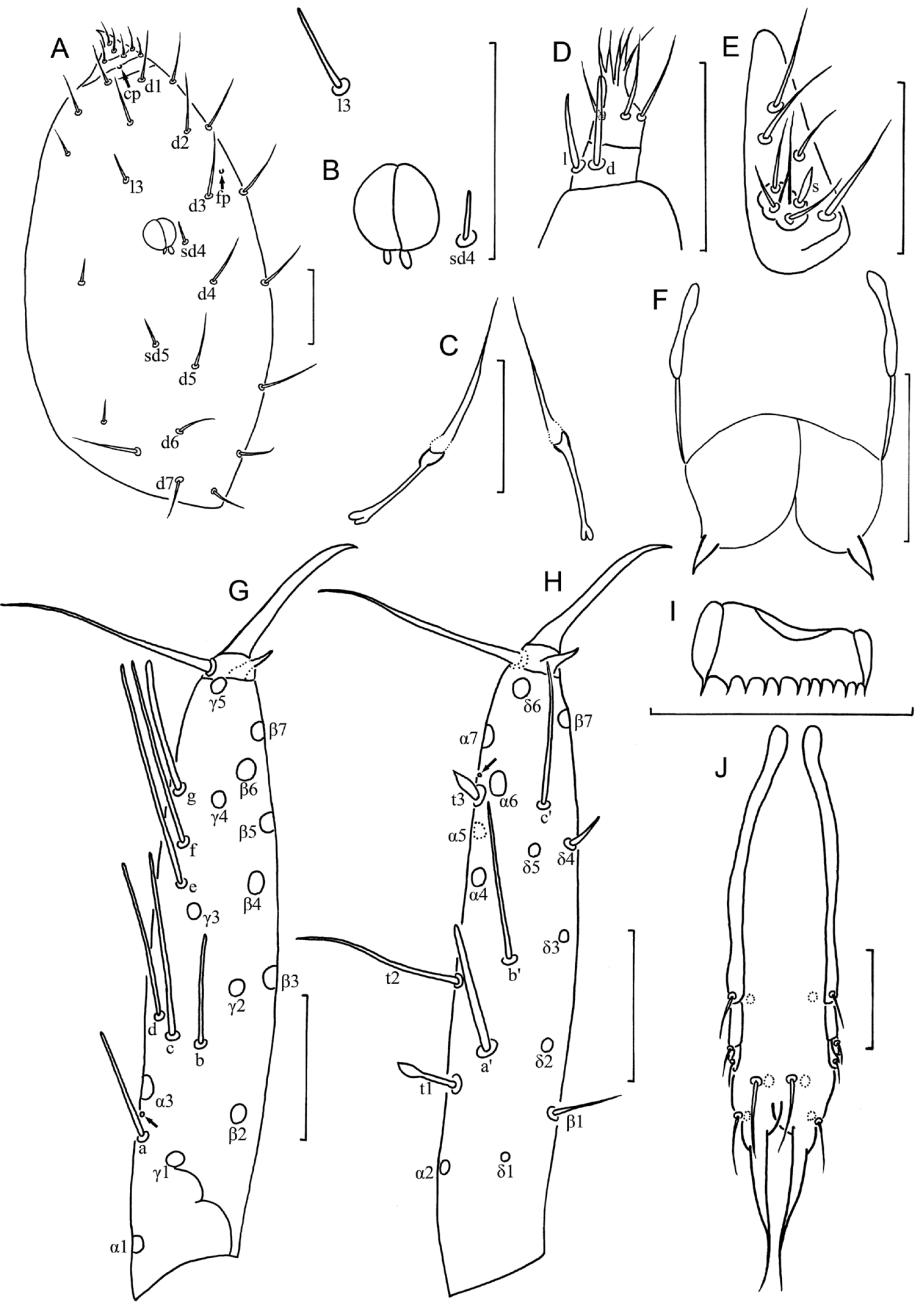
*Yichunentulus alpatovi* sp. n. holotype. **A** Head, dorsal view **B** pseudoculus **C** canal of maxillary gland **D** maxillary palpus **E** labial palpus **F** female squama genitalis **G** foretarsus, exterior view **H** foretarsus, interior view **I** comb **J** male squama genitalis. Arrows indicate pores. Scale bar: 20 μm.

**Head** (Fig. 6A). Ovate, length 120–130 µm, width 70–80 µm. Setae *d6* present, *sd4* and *sd5* short sensilliform. Setae *d6* 11 µm, *d7* 10 µm and *sd7* 18 µm in length. Clypeal pore *cp* and frontal pore *fp* present. Pseudoculus round, length 7–8 µm, with short posterior extension, PR=16 (Fig. 6B). Calyx of maxillary gland smooth, without any appendix, blind end split into two leaves, posterior filament 16–17 µm, CF=7–8 (Fig. 6C). Maxillary palpus with two tapering sensilla, dorsal snsillum is evidently longer than lateral one (Fig. 6D). Labial palpus completed, with one-branched terminal tuft of setae, with three setae and one lanceolate basal sensillum (Fig. 6E).

**Foretarsus** (Fig. 6G, H). Length 86–93 µm, claw length 20–23 µm, without inner flap, TR=4; empodium length 4 µm, EU=0.18. Dorsal sensilla *t-1* baculiform, *t-2* slender and long (23 µm), BS=0.5, *t-3* lanceolate. Exterior sensilla *a* surpassing base of *b* and *c*, *b* slender and short (16 µm), not reaching base of *γ3*, *c* reaching base of *f* and in subequal level to *b*, *d* located higher than *b* and *c*, *e* long and reaching base of claw (30 µm), *f* slender, *g* broad and short (18 µm). Interior sensilla *a*’ broad, *b*’ slender and reaching base of α6, *c*’ nearly reaching base of claw. Relative length of sensilla: *t3* < *t1 < b* < (*a* = *a*’) < *g* < (*b*’ = *c*’) < *d* < *t2* < *c* < *e* < *f*. Setae *β1* setiform and *δ4* sensilliform. Pores close to sensilla *a* and *t3* present. Length of middle tarsus 40 µm, claw length 17 µm. Length of hind tarsus 45 µm, claw length 20 µm.

**Thorax.** Thoracic chaetotaxy given in Table 3. Setae *1* and *2* on pronotum 20 µm and 12–14 µm length respectively. Mesonotum and metanotum with eight pairs of *P*-setae, accessory setae short sensilliform, 2 µm in length; setae *P1*, *P1a* and *P2* on mesonotum 17–20 µm, 3 µm and 20–24 µm respectively. Prosternum with two pairs of anterior seta, and setae *A2* and *M2* sensilliform. Mesosternum and metasternum each with 7 *A*-setae, and setae *A2* sensilliform. Pronotum and prosternum without pores. Mesonotum with pores *sl* and *al*, metanotum with pores *sl* only. Mesosternum and metasternum each with single median pore, situated anterioral to level of setae *M*.

**Table 3.** Adult chaetotaxy of *Yichunentulus alpatovi* sp. n.

Segment		Dorsal		Ventral	
		Formula	Setae	Formula	Setae
Th.	I	4	1, 2	(4+4)/6	A1, 2, M1, 2 P1, 2, 3
	II–III	6/16	A2, 4, M P1, 1a, 2, 2a, 3, 4, 5, 5a	(7+2)/4	Ac, 2, 3, 4, M Pc, 1, 2
Abd.	I	6/12	A1, 2, 5 P1, 1a, 2, 2a, 3, 4	3/4	Ac, 2 P1, 2
	II–III	6/16	A1, 2, 5 P1, 1a, 2, 2a, 3, 4, 4a, 5	3/5	Ac, 2 Pc, 1a, 2
	IV–V	6/16	A1, 2, 5 P1, 1a, 2, 2a, 3, 4, 4a, 5	3/8	Ac, 2 P1, 1a, 2, 3
	VI	8/16	A1, 2, 4, 5 P1, 1a, 2, 2a, 3, 4, 4a, 5	3/8	Ac, 2 P1, 1a, 2, 3
	VII	8/16	A1, 2, 4, 5 P1, 1a, 2, 2a, 3, 3a, 4, 4a, 5	3/8	Ac, 2 P1, 1a, 2, 3
	VIII	6/16	A2, 4, 5 M1, 2, 3, 4, P2, 3, 4, 5	4/2	A1, 2 P1
	IX	14	1, 1a, 2, 2a, 3, 3a, 4	4	1, 2
	X	12	1, 1a, 2, 2a, 3, 4	4	1, 2
	XI	6	1, 3, 4	6	1, 2
	XII	9		6	

**Abdomen.** Abdominal chaetotaxy given in Table 3. Tergite I with three pairs of anterior setae (*A1*, *A2*, *A5*). Tergites II–VI with eight pairs of posterior setae. Tergites VI and VII with four pairs of anterior setae (*A1*, *A2*, *A4*, *A5*). Tergite VII with nine pairs of posterior setae, *P3a* present. All accessory setae on tergites I–VII short sensilliform, 3 µm on tergites I–V, 4 µm on tergite VI, and 5 µm on VII. Tergite VIII with paired setae *M1*. Sternite IV–VII each with eight posterior setae, *Pc* absent. Sternite VIII with two rows of setae (4/2).

Tergites I and VIII with pores *psm* only, II–V with pores *psm* and *al*, VI–VII with pores *psm*, *al* and *psl*, IX–XI without pores, XII with single medial pore. Sternites I–IV without pores, V and VI each with 1+1 posterior pores anterior to seta *P1* and 1+1 anteromembranal pores, VII with single posterior pore asymmetrical located left or right and 1+1 anteromembranal pores, VIII–XI without pores, XII with 1+1 pores *al*.

Abdominal appendages I, II, III with 2, 1, 1 segments and 4, 2, 2 setae respectively. On appendages II and III, subapical setae 16–17 µm, apical setae 13–14 µm in length. Striate band on abdominal segment VIII reduced, anterior margin regular wave shaped. Comb on abdomen VIII rectangular, with 12–13 teeth (Fig. 6I). Female squama genitalis robust, with moderate basal apodeme and pointed acrostyli (Fig. 6F). Male squama genitalis with 5+5 setae on dorsal side and 3+3 setae on ventral side (Fig. 6J).

###### Etymology.

The species is named after Dr. V. Alpatov who accompanied us during our scientific trips.

###### Distribution.

Known only from type locality.

###### Diagnosis.

*Yichunentulus alpatovi* sp. n. is characterized by the presence of sensillum *b*’ on foretarus and short sensilum *b* on foretarsus, 6 anterior seate on tergites IV–V, swelled sensillum *a*’, presence of pores *psl* only on tergites VI and VII, and female squama genitalis with moderate basal apodeme and pointed acrostyli.

###### Remarks.

The present species is located in the genus *Yichunentulus* because the labial palpus with one-branched terminal tuft of setae, with three setae and one lanceolate basal sensillum, the baculiform sensillum *t1* on foretarus, reduced striate band, two pairs of anterior setae on mesonotum and metanotum, 4/2 setae on sternite VIII, and abdominal appendages II and III each with two setae of different length. *Yichunentulus alpatovi* sp. n. is close to the type species *Yichunentulus yichunensis* Yin, 1980 in having short sensillum *b* and identical body chaetotaxy. However, it can be easily distinguished from *Yichunentulus yichunensis* and *Yichunentulus borealis* (Nakamura, 2004), comb. n. by the presence of sensillum *b*’ on foretarsus. It also differs from *Yichunentulus yichunensis* in the length of sensillum *e* (extremely long in *Yichunentulus alpatovi* sp. n. vs. short in *Yichunentulus yichunensis*) and sensilla *c*’ (long and surpassing base of *δ6* in *Yichunentulus alpatovi* sp. n. vs. short and only reaching base of *β7* in *Yichunentulus yichunensis*). It differs from *Yichunentulus borealis* in the chaetotaxy of tergites IV–V (each with 6 *A*-setae in *Yichunentulus alpatovi* sp. n. vs. 8 in *Yichunentulus borealis*), shape of sensillum *a*’ (slightly broad and as long as sensillum *a* in *Yichunentulus alpatovi* sp. n. vs. distinctly swell and shorter than sensillum *a* in *Yichunentulus borealis*), and the body porotaxy (pore *psl* present on tergites VI and VII only and sternites I–IV without pores in *Yichunentulus alpatovi* sp. n. vs. *psl* present on tergites III–VII and sternites II and IV each with 1+1 anteromembranal pores in *Yichunentulus borealis*).

#### New records of genera, keys and new taxonomic combinations

##### Genus *Acerentulus* Berlese, 1908, new to RFE

###### Acerentulus

Taxon classificationAnimaliaProturaAcerentomidae

Berlese, 1908: 122.

####### Type species.

*Acerentomon confine* Berlese, 1908.

####### Diagnosis.

Abdominal appendages II and III each with 3 setae, mesonotum and metanotum each with two pairs of anterior setae, foretarsal sensillum *t1* claviform, sensillum *b*’ present, *t3* willow-leaf shaped, sensillum *b*’ present, labial palpus with terminal tuft of setae, striate band on abdominal segment VIII well developed, and sternite VIII with 4/2 setae or with 4 setae only.

####### Remarks.

The genus *Acerentulus* has 47 species described in the world and most from Europe (Szeptycki 2007; Shrubovych et al. 2012), and only five species (*Acerentulus kisonis* Imdadaté, 1961, *Acerentulus keikoae keikoae* Imdadaté, 1988, *Acerentulus keikoae capillatus* Imdadaté, 1988, *Acerentulus omoi* Imdadaté, 1988 and *Acerentulus sinensis* Wu & Yin, 2007) were recorded in East Asia so far. *Acerentulus kisonis* Imdadaté, 1961 is new to RFE and very rare in the samples like in Japan (Imdadaté 1988), which has single individual present in hundreds of specimens.

##### Genus *Fjellbergella* Nosek, 1978 neto RFE

###### Fjellbergella

Taxon classificationAnimaliaProturaAcerentomidae

Nosek, 1978: 57.

####### Type species.

*Fjellbergella tuxeni* Nosek, 1980.

####### Diagnosis.

Mesonotum and metanotum each with two pairs of anterior setae, labial palpus with two-branched terminal tuft of setae, sensillum *d* located near *t2* insertion, abdominal appendages II and III each with three setae, foretarsal sensillum *t1* claviform, sensillum *b*’ present, striate band on abdominal segment VIII reduced and sternite VIII with 4/2 setae.

####### Remarks.

The genus *Fjellbergella* containes only two species so far: *Fjellbergella tuxeni* Nosek, 1980 from Alaska and *Fjellbergella uteorum* Shrubovych & Bernard, 2013 from Colorado (Nosek 1980; Shrubovych and Bernard 2013). Except that, one species of the similar form *Brasilidia jilinensis* Wu & Yin, 2007 was also found from Northeast China. However, the species of genus *Brasilidia* has reduced labial palpus without tuft, and all of them occurred in tropical area (South America and India). After recheck the type specimens, we confirmed that *Brasilidia jilinensis* has reduced striate band, the labial palpus with reduced tuft (two-brunched), and three setae on abdominal appendages II and III which indicate it is a member of *Fjellbergella*. Thus we transfer *Brasilidia jilinensis* to the genus *Fjellbergella* as a new combination *Fjellbergella jilinensis* (Wu & Yin, 2007), comb. n. Plus the new species described in present paper, the genus *Fjellbergella* contains 4 species. They can be distinguished by the following key.

####### Key to the species of genus *Fjellbergella* Nosek, 1978

**Table:** 

1	Tergite VII with 4 pairs of anterior setae, seta *Pc* absent	2
–	Tergite VII with 5 pairs of anterior setae, seta *Pc* present	*Fjellbergella uteorum* Shrubovych & Bernard, 2013; USA (Colorado)
2	Tergites I–VI without seta *P1a*	*Fjellbergella jilinensis* (Wu & Yin, 2007), comb. n.; China (Jilin)
–	Tergites I–VI with seta *P1a*	3
3	Tergites IX and X with 14 and 12 setae respectively, sensillum *b* shorter than *c* and they located at the same level, claw of foreleg without inner flap	*Fjellbergella lazovskiensis* sp. n.; Russia (Far East)
–	Tergites IX and X with 12 and 8 setae respectively, sensillum *b* and *c* subequal in length and *c* located lower than *b* distinctly, claw of foreleg with one inner flap	*Fjellbergella tuxeni* Nosek, 1980; USA (Alaska)

###### Fjellbergella jilinensis

Taxon classificationAnimaliaProturaAcerentomidae

(Wu & Yin, 2007) comb. n.

Brasilidia jilinensis Wu & Yin, 2007: 53–61, figs 19–36. **Syn.**

####### Material examined.

Holotype, female, Paratype, 1 female, China, Jilin Province, Dehui city, 15-IX-2006, coll. D. H. Wu.

####### Distribution.

China (Jilin).

##### Genus Yichunentulus

Taxon classificationAnimaliaProturaAcerentomidae

Yin, 1980 new to RFE

Yichunentulus Yin, 1980: 146-147, 155.

###### Type species.

*Yichunentulus yichunensis* Yin, 1980.

###### Diagnosis.

Mesonotum and metanotum each with two pairs of anterior setae, abdominal appendages II and III each with two setae, labial palpus with one-branched terminal tuft of setae, with 3 setae and 1 sensillum, foretarsal sensillum *t1* baculiform, sensillum *b*’ absent or present, sensillum *a*’ located distal to *t1*, maxillary gland simple and without appendages, sternite VIII with 4/2 setae, striate band on abdominal segment VIII reduced, and sternites II–VI each has 1+1 membranal pores.

###### Remarks.

The genus *Yichunentulus* Yin, 1980 has only one species described from Heilongjiang, Northeast China (Yin 1980). After compare *Baculentulus borealis* Nakamura, 2004 with congeners, we find two important characters of this species: presence of one-branched terminal tuft of setae and 3 seate on labial palpus and 4/2 setae on sternite VIII are different to any other species of the genus *Baculentulus*. On the contrary, those two characters match well with genus *Yichunentulus*. Two specimens of *Baculentulus borealis* newly collected from type locality are also studied. We proposed to transfer *Baculentulus borealis* to the genus *Yichunentulus* as a new combination *Yichunentulus borealis* (Nakamura, 2004), comb. n. The three species of the genus *Yichunentulus* can be distinguished by the following key.

###### Key to species of genus *Yichunentulus* Yin, 1980

**Table:** 

1	Tergites IV and V each with 4 pairs of anterior setae	*Yichunentulus borealis* (Nakamura, 2004), comb. n.
–	Tergites IV and V each with 3 pairs of anterior setae	2
2	Foretarsal sensillum *b*’ absent	*Yichunentulus yichunensis* Yin, 1980
–	Foretarsal sensillum *b*’ present	*Yichunentulus alpatovi* sp. n.

##### Yichunentulus borealis

Taxon classificationAnimaliaProturaAcerentomidae

(Nakamura, 2004) comb. n.

Baculentulus borealis Nakamura, 2004: 17–20, figs 1–15. **Syn.**

###### Material examined.

1 female, 1 male, Locality 3, 25-IV-2010, coll. E. Sokolova & M. Potapov.

###### Distribution.

Russia (Far East, Khabarovsk Krai).

###### Notes.

Body length 1300–1380 µm, foretarsus length 110 µm. We studied the new materials of *Yichunentulus borealis* collected from type locality and redescribe the head chaetotaxy and body porotaxy. Head with *d6* seta present, *sd4* and *sd5* sensilliform. Pronotum and prosternum without pores. Mesonotum with pores *sl* and *al*, metanotum with pores *sl* only. Mesosternum and metasternum each with single medial pore. Tergites I and VIII with pores *psm* only, II with pores *psm* and *al*, III–VII with pores *psm*, *al* and *psl*, IX–XI without pores, XII with single medial pore. Sternites I with single medial pore, II–IV without 1+1 anteromembranal pores, V and VI each with 1+1 posterior pores anterior to seta *P1* and 1+1 anteromembranal pores, VII with single posterior pore asymmetrical located left or right, VIII–XI without pores, XII with 1+1 pores *al*.

#### Description of known species

##### Imadateiella sharovi

Taxon classificationAnimaliaProturaNipponentomidae

(Martynova, 1977)

**Figure 7.**
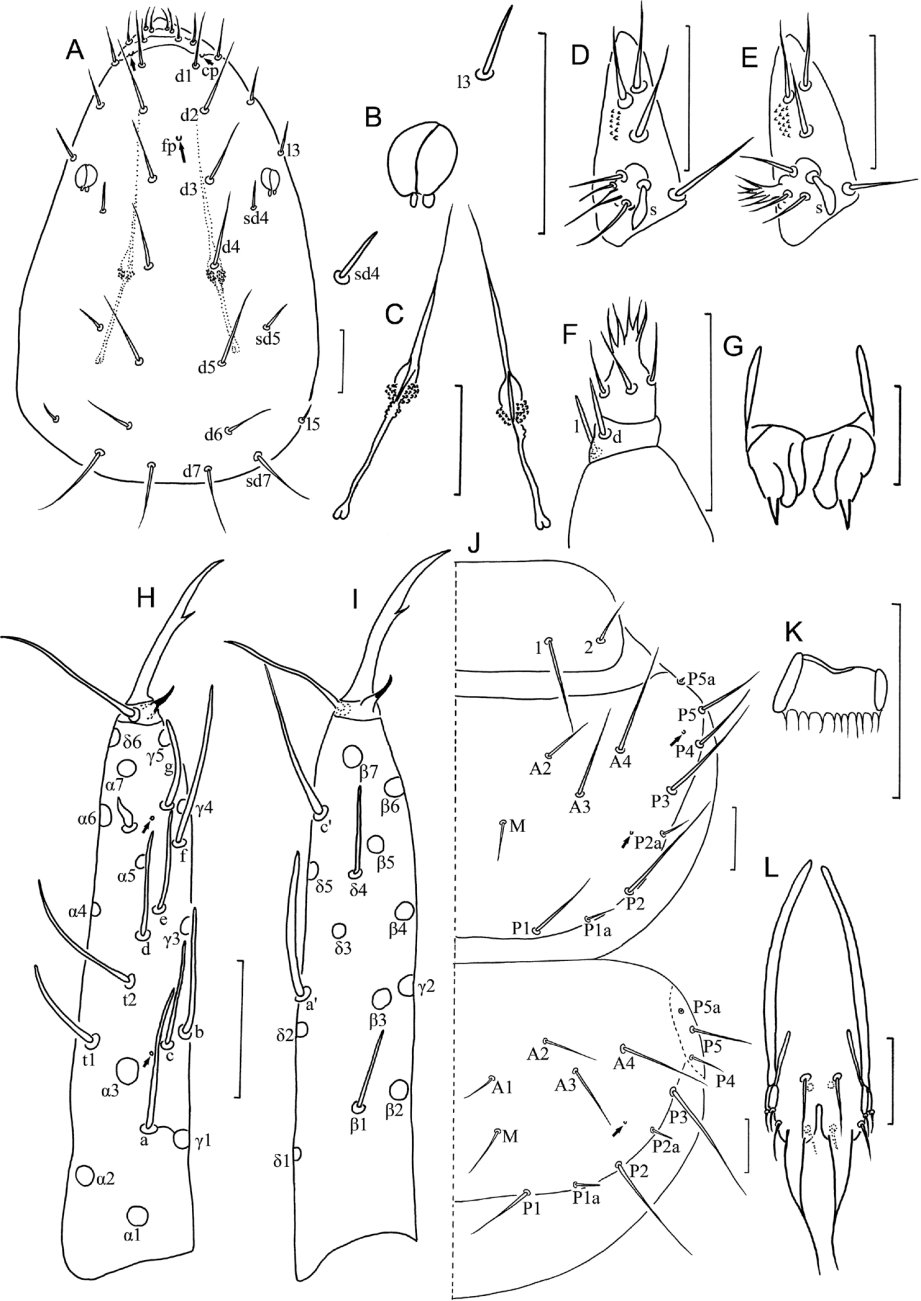
*Imadateiella sharovi* (Martynova, 1977) **A** Head, dorsal view **B** pseudoculus **C** canal of maxillary gland **D** labial palpus (specimens from samples 24 and 27) **E** labial palpus (specimens from samples 26, 30 and 32) **F** maxillary palpus **G** female squama genitalis **H** foretarsus, exterior view **I** foretarsus, interior view **J** nota, right side **K** comb **L** male squama genitalis. **A–D** and **F–L** specimen No. FE-2011062-4; **E** specimen No. FE-2011060-1. Arrows indicate pores. Scale bar: 20 μm.

**Figure 8.**
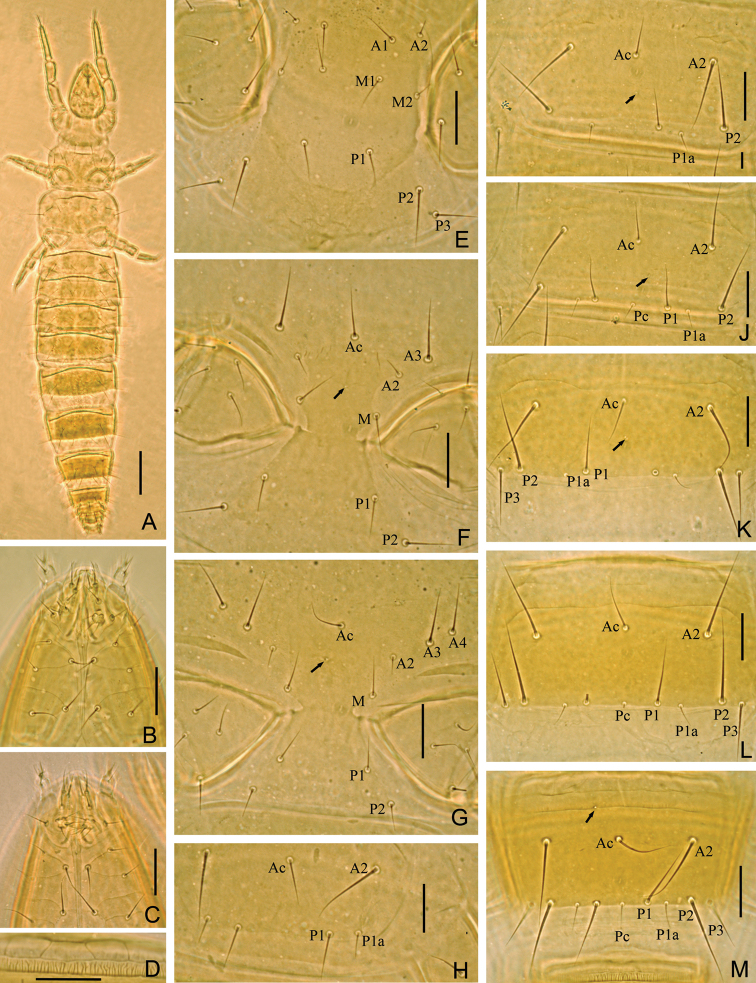
*Imadateiella sharovi* (Martynova, 1977) **A** Habitus **B** ventral side of head, shows reduced labial palpus **C** ventral side of head, shows well developed labial palpus **D** part of striate band **E** prosternum **F** mesosternum **G** metasternum **H–M** sternites I–III, IV, VI and VII. **A–B** and **D–M** specimen No. FE-2011062-4; **C** specimen No. FE-2011060-1. Arrows indicate pores. Scale bar: 100 μm in (**A**), others, 20 μm.

Figs 7 , 8 ; Table 4

Acerella sharovi Martynova, 1977: 164–166, Figs 1, 2. **Syn.**
Imadateiella sharovi (Martynova, 1977), Imadaté 1981: 144.

###### Material examined.

7 females (Nos. FE-2011062-1, FE-2011062-4, FE-2011062-5, FE-2011085-1, FE-2011085-2, FE-2011087-1, FE-2011089-1) (SEM; MSPU), 5 males (Nos. FE-2011062-2, FE-2011062-3, FE-2011086-1, FE-2011086-2, FE-2011087-2) (SEM; MSPU), 1 male preimago (No. FE-2011090-2), Russia, Far East, Khabarovsk Krai, Vaninsky area, nearby Vysokogorny, Valley of Mulinka, from *Picea* and *Abies* forest on Northeast slope, sample No. 27, 750 m alt., 30-IX-2011, 1 male preimago (No. FE-2011059-2), 1 maturus junior (No. FE-2011059-1), Russia, Far East, Khabarovsk Krai, Vaninsky area, nearby Datta, from coastal larch-wood on East slope (green moss), sample No. 24, 28-IX-2011. 1 female (No. FE-2011060-1), Russia, Far East, Khabarovsk Krai, Vaninsky area, nearby Vysokogorny, Valley of Mulinka, from spruce forest at pass, sample No. 26, 900 m alt., 29-IX-2011. 2 females (FE-2011067-2, FE-2011088-1), 1male (No. FE-2011088-2), Russia, Far East, Khabarovsk Krai, Vaninsky area, nearby Vysokogorny, Valley of Mulinka, from mixed sample of spruce-forest and rotten wood, sample No. 30, 600 m alt., 29-ix-2011. 1 female (No. FE-2011069-1), Russia, Far East, Khabarovsk Krai, Vaninsky area, nearly Vysokogorny (5 km South), Valley of Dopolnitel’ny Stream, from mixed sample of litter under old poplar-trees in floodplain, sample No. 32, 400 m alt., 30-IX-2011. All specimens are collected by M. Potapov & V. Alpatov.

###### Description of new materials.

Adult body length 1000–1200 µm (n=16), yellow in color (Fig. 8A).

**Head** (Fig. 7A). Ovate, length 130–135 µm, width 80–85 µm. Setae *d6* present, *sd4* and *sd5* short. Setae *d6* 14–15 µm, *d7* 16–17 µm and *sd7* 18–19 µm in length. Clypeal pore *cp* and frontal pore *fp* present. Pseudoculus round, length 7–8 µm, with short posterior extension, PR=16–17 (Fig. 7B). Maxillary gland large, calyx with lateral racemose appendices and one helmet-like dorsal appendix, and bilobed posterior dilation, posterior filament length 20–22 µm, CF=6–7 (Fig. 7C). Labial palpus reduced, with one-branched terminal tuft of setae, three setae and one leaf-shaped basal sensillum (Figs 7D, 8B), but well developed in five specimens (Figs 7E, 8C). Maxillary palpus with two tapering sensilla, subequal in length (Fig. 7F).

**Foretarsus** (Fig. 7H, I). Length 85–92 µm, claw length 23–29 µm, with one inner flap, TR=3.5–3.7; empodium length 4 µm, EU=0.15–0.18. Dorsal sensilla *t-1* filiform, *t-2* slender and long (17 µm), BS=0.33, *t-3* lanceolate and short. All exterior sensilla slender, *a* far surpassing base of *b* and *c*, *b* slightly longer than *c*, *c* short and lower than *b*, *d* and *e* short, *f* long, *g* broad and short. Interior sensilla *a*’ broad and long, surpassing base of *δ5*, *b*’ absent, *c*’ slender and surpassing base of claw. Relative length of sensilla: *t3* < *g* < *t1* < (*c* = *d* = *e*) < *b* < *a*’ < *a* < *t2* < *c*’ < *f*. Setae *β1* and *δ4* sensilliform, 12–13 µm in length. Pores close to sensilla *c* and *t3* present. Length of middle tarsus 40 µm, claw length 15–20 µm. Length of hind tarsus 45 µm, claw length 18–20 µm.

**Thorax.** Thoracic chaetotaxy given in Table 4. Setae *1* and *2* on pronotum 26–35 µm and 16–17 µm length respectively, mesonotum and metanotum with eight pairs of *P*-setae, accessory setae sensilliform; setae *P1*, *P1a* and *P2* on mesonotum 30 µm, 10 µm and 43 µm respectively (Fig. 7J). Prosternum with two pairs of anterior seta, and setae *A2* and *M2* sensilliform (Fig. 8E). Mesosternum with 5 *A*-setae, and metasternum each with 7 *A*-setae, and setae *A2* sensilliform (Fig. 8F, G). Pronotum and prosternum without pores (Figs 7J, 8E). Mesonotum with pores *sl* and *al*, metanotum with pores *sl* only (Fig. 7J). Mesosternum and metasternum each with single medial pore (Fig. 8F, G). Single membranal pore present on membrane between each coxa and the body.

**Table 4.** Adult chaetotaxy of *Imadateiella sharovi* (Martynova, 1977).

Segment		Dorsal		Ventral	
		Formula	Setae	Formula	Setae
Th.	I	4	1, 2	(4+4)/6	A1, 2, M1, 2 P1, 2, 3
	II	8/16	A2, 3, 4, M P1, 1a, 2, 2a, 3, 3a, 4, 5	(5+2)/4	Ac, 2, 3, M P1, 2
	III	10/16	A1, 2, 3, 4, M P1, 1a, 2, 2a, 3, 3a, 4, 5	(7+2)/4	Ac, 1, 2, 3, M P1, 2
Abd.	I	6/12	A1, 2, 5 P1, 1a, 2, 2a, 3, 4	3/4	Ac, 2 P1, 1a
	II	10/16	A1, 2, 3, 4, 5 P1, 1a, 2, 2a, 3, 4, 4a, 5	3/5	Ac, 2 Pc, 1a, 2
	III	10/16	A1, 2, 3, 4, 5 P1, 1a, 2, 2a, 3, 4, 4a, 5	3/6	Ac, 2 P1, 1a, 2
	IV–V	10/16	A1, 2, 3, 4, 5 P1, 1a, 2, 2a, 3, 4, 4a, 5	3/8	Ac, 2 P1, 1a, 2, 3
	VI	10/16	A1, 2, 3, 4, 5 P1, 1a, 2, 2a, 3, 4, 4a, 5	3/9	Ac, 2 Pc, 1, 1a, 2, 3
	VII	8/16	A2, 3, 4, 5 P1, 1a, 2, 2a, 3, 4, 4a, 5	3/9	Ac, 2 Pc, 1, 1a, 2, 3
	VIII	6/15	A1, 3, 5 Mc, 2, 3, 4, P2, 3, 4, 5	4/2	1, 2 1a
	IX	12	1, 1a, 2, 2a, 3, 4	4	1, 2
	X	10	1, 2, 2a, 3, 4	4	1, 2
	XI	6	1, 3, 4	6	1, 2, 3
	XII	9		6	

**Abdomen.** Abdominal chaetotaxy given in Table 4. Tergite I with three pairs of anterior setae (*A1*, *A2*, A5) and six pairs of posterior setae. Tergites II–VII with eight pairs of posterior setae, *P3a* present. Tergite VII with four pairs of anterior setae (*A2*, *A3*, *A4*, *A5*). Tergite VII with nine pairs of posterior setae. All accessory setae on tergites I–VII sensilliform, 14–15 µm on tergites I–VI and 17 µm on VII. Tergite VIII with setae *Mc*). Sternite IV–V each with three anterior setae and eight posterior setae (Fig. 8K), VI–VII each with three anterior setae and nine posterior setae, *Pc* present (Fig. 8L, M). Sternite VIII with 4/2 setae. Hind margin of tergite IX smooth. Hind margin of tergites X–XI with very short fine ciliation. Hind margin of sternites IX–XI smooth. Posterior margin of tergite XII smooth, sternite with delicate serration in central part.

Tergites I and VIII with pores *psm* only, II–VII with pores *psm* and *al*, IX–XI without pores, XII with single medial pore. Sternites I and VI without pores (Fig. 8H, L), II–V each with single medial pore (Fig. 8I, J), VII with single anterior pore asymmetrical located left or right on the line (Fig. 8M), VIII–XI without pores, XII with 1+1 pores *al*.

Abdominal appendages I, II, III with 2, 1, 1 segments and 4, 2, 2 setae respectively. On appendages II and III, subapical seta 16–18 µm, apical seta 12–15 µm in length. Striate band on abdominal segment VIII well developed, anterior margin regular wave shaped (Fig. 8D). Comb on abdomen VIII rectangular, with 10–12 teeth (Fig. 7K). Female squama genitalis with short basal apodeme and pointed acrostyli (Fig. 7G). Male squama genitalis with 4+4 setae on dorsal side and 2+2 setae on ventral side (Fig. 7L).

**Chaetal variability.** Chaetal variations were observed in 7 specimens: on tergite II, absence of *P4a* (No. FE-2011067-2); on tergite IV, asymmetrical absence of *A1* of right side (No. FE-2011060-1); on sternite II, absence of *Pc* and present of P1 on right side (No. FE-2011060-1, Fig. 70); on sternite III, present of *Pc* (No. FE-2011060-1, Fig. 71); on sternite VI, absence of *Pc* (Nos. FE-2011067-2, FE-2011088-2, FE-2011089-1); on sternite VIII, asymmetrical absence of *P1* of left side (Nos. FE-2011062-3, FE-2011062-5, FE-2011069-1), or both side (No. FE-2011060-1).

###### Distribution.

Russia (Far East, Magadan Oblast; Khabarovsk Krai).

###### Diagnosis.

*Imadateiella sharovi* (Martynova, 1977) is characterized by the presence of setae *Pc* on sternite VI and 4/2 setae on sternite VIII.

###### Remarks.

*Imadateiella sharovi* (Martynova, 1977) is the first species of Protura described in RFE. We give the redescription of *Imadateiella sharovi* basing on our vast material since it shows minor differences from the redescription of Shrubovych (2014) (labial palpus, length of sensilla *b*, *e* and *t2* on fortarsus and pores on sternite I). The male squama genitalis is also described for the first time. The variation on labial palpus is also uncommon – 15 specimens from samples 27 and 24 have labial palpus with reduced terminal tuft of setae (Figs 7D, 8B) while other 5 specimens from samples 26, 30 and 32 have it well developed (Figs 7E, 8C). According to the redescription of Shrubovych (2014), the type specimens have well developed labial palpus. We treat all our populations as belonging to one variable species.

#### List of species of Russian Far East

##### Family Berberentulidae Yin, 1983

###### *Baculentulus krabbensis* sp. n.

The description is given above.

###### Baculentulus loxoglenus

Taxon classificationAnimaliaProturaBerberentulidae

Yin, 1980

####### Material examined.

3 females, locality 4, 10-IX-2011, coll. Y. Bu, C. W. Huang, M. Potapov & V. Alpatov.

####### Distribution.

Northeast China; Russia (Far East: Khabarovsk Krai; Primorsky Krai). For Russia, it was already recorded from Khabarovsk Krai by Nakamura (2004) and we found it from Primorsky Krai.

###### Baculentulus morikawai

Taxon classificationAnimaliaProturaBerberentulidae

(Imadaté & Yosii, 1956)

####### Material examined.

2 females, 2 males, locality 3, 25-IV-2010, E. Sokolova & M. Potapov collected; 2 females, 5 males, locality 5, 16-IX-2011, coll. Y. Bu, C. W. Huang, M. Potapov & V. Alpatov; 36 females, 39 males, 5 male preimagos, locality 7, 22-IX-2011, coll. Y. Bu, C. W. Huang, M. Potapov & V. Alpatov.

####### Distribution.

Widely distributed in eastern areas of Asia: China (Anhui, Taiwan, Xianggang, Yunnan, Zhejiang); Japan; Korea; Russia (Far East: Khabarovsk Krai: Khekhtsyr Range; Primorsky Krai: Shkotovsky area). New for Russia.

###### Baculentulus samchonri

Taxon classificationAnimaliaProturaBerberentulidae

Imdadaté & Szeptycki, 1976

####### Material examined.

1 female, 1 male, locality 5, 16-IX-2011, 12 females, 7 males, locality 7, 22-IX-2011, coll. Y. Bu, C. W. Huang, M. Potapov & V. Alpatov.

####### Distribution.

Korea; Russia (Far East: Primorsky Krai). New for Russia.

###### Baculentulus potapovi

Taxon classificationAnimaliaProturaBerberentulidae

Shrubovych, 2010

####### Material examined.

3 females, 4 males, 2 maturi juniors, 1 prelarva, locality 4, 10-IX-2011, coll. Y. Bu, C. W. Huang, M. Potapov & V. Alpatov.

####### Distribution.

Russia (Far East: Primorsky Krai). It was described from Partizansky, Shkotovsky and Khasansky areas by Shrubovych (2010) and we found it from the same areas.

##### Family Acerentomidae Silvestri, 1907

###### Acerentulus kisonis

Taxon classificationAnimaliaProturaAcerentomidae

Imdadaté, 1961

####### Material examined.

1 female, locality 7, 21-IX-2011. coll. Y. Bu, C. W. Huang, M. Potapov & V. Alpatov.

####### Distribution.

Japan; Russia (Far East: Primorsky Krai). New for Russia.

####### Notes.

Body large and robust, 1700 µm in length. Foretarsus length 115 µm, setae *β1* and *δ4* sensilliform, 5 µm in length. Setae *d6* present on head, 16 µm. Clypeal pore *cp* and frontal pore *fp* present. Pronotum and prosternum without pores, mesonotum with pores *sl* and *al*, metanotum with pores *sl*. Mesosternum and metasternum each with 1 medial pore. Tergites I and VIII with pores *psm* only, II–V with pores *psm* and *al*, VI–VII with pores *psm*, *al*, and *psl*. X–XI without pores, XII with single medial pore. Pores on sternites I–IV and VII not observed. Sternite V with 1 posterior pore. Sternite VI with 4 posterior pores composed by two groups, each group contains two close adjacent pores. VIII–XI without pores, XII with 1+1 pores *al*.

###### Filientomon gentaroanum

Taxon classificationAnimaliaProturaAcerentomidae

Nakamura, 2001

####### Material examined.

1 female, 2 maturi juniors, locality 5, 16-IX-2011. coll. Y. Bu, C. W. Huang, M. Potapov & V. Alpatov.

####### Distribution.

Japan; Russia (Far East: Primorsky Krai). New for Russia.

###### Filientomon takanawanum

Taxon classificationAnimaliaProturaAcerentomidae

(Imdadaté, 1956)

####### Material examined.

1 female, 2 maturi juniors, locality 4, 9-IX-2011, coll. Y. Bu, C. W. Huang, M. Potapov & V. Alpatov; 5 females, 2 males, 3 maturi juniors, 2 Larvae II, locality 2, 30-ix-2011, coll. M. Potapov & V. Alpatov.

####### Distribution.

Widely distributed in Asia: China (Anhui, Hebei, Jilin, Shanxi, Zhejiang); Japan; Korea; Russia (Far East: Primorsky Krai; Khabarovsk Krai). New for Russia.

###### *Fjellbergella lazovskiensis* sp. n.

The description is given above.

###### Tuxenentulus ohbai

Taxon classificationAnimaliaProturaAcerentomidae

Imdadaté, 1974

####### Material examined.

1 female, 1 male, locality 2, sample No. 29, 30-IX-2011, coll. M. Potapov & V. Alpatov.

####### Distribution.

Northeast China; Japan; Russia (Far East: Khabarovsk Krai). New for Russia.

###### Yamatentomon yamato

Taxon classificationAnimaliaProturaAcerentomidae

Imadaté & Yosii, 1956

####### Material examined.

4 females, 2 males, 2 maturi juniors, 1 Larva LII, locality 3, 20-IX-2009. coll. O. Smirnova; 2 females, 1 larva I, 1 larva II, locality 3, 24-IV-2010, coll. E. Sokolova & M. Potapov; 2 females, 3 males, 2 larvae II, locality 4, 10-IX-2011; 3 females, 1 males, 1 maturus junior, locality 5, 16-IX-2011; 5 females, 4 males, 3 maturi juniors, 3 larvae II, 1 males preimago, locality 7, 22-IX-2011. coll. Y. Bu, C. W. Huang, M. Potapov & V. Alpatov.

####### Distribution.

Northeast China; Japan; Korea; Russia (Far East: Primorsky Krai; Khabarovsk Krai). It was reported from Shkotovsky and Khasansky areas by Shrubovych (2014) and we found it from the same areas.

###### *Yichunentulus alpatovi* sp. n.

The description is given above.

###### *Yichunentulus borealis* (Nakamura, 2004), comb. n.

The description is given above.

##### Family Nipponentomidae Yin, 1996

###### Callientomon chinensis

Taxon classificationAnimaliaProturaNipponentomidae

Yin, 1980

####### Material examined.

5 females, locality 7, 22-IX-2011, coll. Y. Bu, C. W. Huang, M. Potapov & V. Alpatov.

####### Distribution.

Northeast China; Russia (Far East: Primorsky Krai, Lazovsky area). It was reported from Shkotovsky and Khasansky areas by Shrubovych (2014) and we also found it from Lazovsky area.

###### Verrucoentomon louisanne

Taxon classificationAnimaliaProturaNipponentomidae

Shrubovych, 2012

####### Material examined.

4 females, locality 4, 10-IX-2011, coll. Y. Bu, C. W. Huang, M. Potapov & V. Alpatov.

####### Distribution.

Russia (Far East, Primorsky Krai). It was recorded from Ussuriysky, Khasansky and Shkotovsky areas by Shrubovych and Bernard (2012) and we found it from Shkotovsky area again.

###### Verrucoentomon shirampa

Taxon classificationAnimaliaProturaNipponentomidae

Imadaté, 1964

####### Material examined.

10 females, 9 males, locality 3, 20-IX-2009. coll. O. Smirnova; 2 females, 1 male, locality 3, 24-IV-2010, E. Sokolova & M. Potapov collected; 1 female, locality 4, 10-IX-2011, Y. Bu, C. W. Huang, M. Potapov & V. Alpatov collected.

####### Distribution.

Japan; Korea; Russia (Far East: Khabarovsk Krai; Prmorsky Krai). It was already recorded from Ussuriysky and Shkotovsky areas by Shrubovych and Bernard (2012) and we found it also occurred in Khabarovsk Krai.

###### *Imadateiella sharovi* (Martynova, 1977)

The description of the new material from Russian Far East is given above.

###### Nipponentomon cf. bidentatum

Taxon classificationAnimaliaProturaNipponentomidae

Nakamura, 2004

####### Material examined.

2 males, locality 3, 20-IX-2009, coll. O. Smirnova; 3 females, 2 males, locality 2, 30-IX-2011, coll. M. Potapov & V. Alpatov.

####### Distribution.

Russia (Far East, Khabarovsk Krai). New for Russia.

####### Notes.

The present species is nearly identical to *Nipponentomon bidentatum* Nakamura, 2004 found from Korfovsky area of Khabarovsk Krai, they have the same shape of sensilla on foretarsus and the body chaetotaxy (seta *P3a* absent on tergites II–VII and *A3* present on tergite I), our form only differs in the presence of seta *d6* on head and 1 inner flap on the claw. Final decision on the status of our population calls for the additional study.

###### Nipponentomon cf. heterothrixi

Taxon classificationAnimaliaProturaNipponentomidae

Yin & Xie, 1993

####### Material examined.

2 males, locality 5, 16-IX-2011, coll. Y. Bu, C. W. Huang, M. Potapov & V. Alpatov.

####### Distribution.

Russia (Far East, Primorsky Krai). New for Russia.

####### Notes.

The present species has nearly the same body chaetotaxy (with *P2a*’ on mesonotum and metanotum) and the shape of sensilla on foretarsus as in *Nipponentomon heterothrixi* described from Northeast China, but differs by presence of setae *P3a* on tergites II–V which are absent in *Nipponentomon heterothrixi*. Insufficient material does not allow describing a new species.

###### Nipponentomon khabarovskense

Taxon classificationAnimaliaProturaNipponentomidae

Nakamura, 2004

####### Material examined.

2 females, 2 males, locality 7, 21-IX-2011, coll. Y. Bu, C. W. Huang, M. Potapov & V. Alpatov. 1 female, 1 male, locality 2, 30-IX-2011, coll. M. Potapov & V. Alpatov.

####### Distribution.

Russia (Far East, Khabarovsk Krai; Primorsky Krai ). New for Russia.

####### Notes.

The present species was described from Korfovsky area of Khabarovsk Krai by Nakamura (2004) and we found it also occurred in Primorsky Krai.

###### Nipponentomon jaceki

Taxon classificationAnimaliaProturaNipponentomidae

Shrubovych, 2009

####### Material examined.

3 females, locality 4, 10-IX-2011, coll. Y. Bu, C. W. Huang, M. Potapov & V. Alpatov.

####### Distribution.

Russia (Far East, Primorsky Krai). It was recorded from Shkotovsky area by Shrubovych (2009) and we found it from the same area again.

###### Nipponentomon nippon

Taxon classificationAnimaliaProturaNipponentomidae

(Yoshii, 1938)

####### Material examined.

1 female, locality 5, 16-IX-2011; 2 females, locality 7, 21-IX-2011. coll. Y. Bu, C. W. Huang, M. Potapov & V. Alpatov.

####### Distribution.

Russia (Far East, Primorsky Krai). It was already recorded from Partizansky and Khasansky areas by Shrubovych (2009). We found it also occurred in Lazovsky area.

##### Family Eosentomidae Berlese, 1909

###### Eosentomon asahi

Taxon classificationAnimaliaProturaEosentomidae

Imadaté, 1961

####### Material examined.

1 male, locality 3, 2-X-2009, coll. O. Smirnova; 5 females, 1 male, 1 larva I, and 1 larva II, locality 4, 10-IX-2011, coll. Y. Bu, C. W. Huang, M. Potapov & V. Alpatov.

####### Distribution.

Russia (Far East: Khabarovsk Krai; Primorsky Krai). It is already recorded from Khabarovsk Krai by Nakamura (2004), and we also found it in Primorsky Krai.

###### Eosentomon brevicorpusculum

Taxon classificationAnimaliaProturaEosentomidae

Yin, 1965

####### Material examined.

1 female, locality 4, 10-IX-2011; 1 female, 1 male, 1 larva I, locality 7, 21-IX-2011, 1 female, 1 male, locality 7, 22-IX-2011; coll. Y. Bu, C. W. Huang, M. Potapov & V. Alpatov.

####### Distribution.

Widely distributed in China. Russia (Far East, Primorsky Krai). New for Russia.

###### Eosentomon orientale

Taxon classificationAnimaliaProturaEosentomidae

Yin, 1965 new to RFE

####### Material examined.

4 females, 1 male, locality 7, 22-IX-2011, coll. Y. Bu, C. W. Huang, M. Potapov & V. Alpatov.

####### Distribution.

Widely distributed in China; Russia (Far East, Primorsky Krai). New for Russia.

## Discussion

The 31 species of Protura recorded from RFE so far are listed in Table 5. They belong to 12 genera and 4 families Berberentulidae, Acerentomidae, Nipponentomidae and Eosentomidae, half the species are known only from the RFE. The most species are representatives of Acerentomidae and Nipponentomidae, each with 11 species (70%). The family Eosentomidae is rare and consists of only 3 species. Palearctic genera *Yamatentomon*, *Imadateiella*, *Yichunentulus*, *Callientomon* and Holarctic genera *Filientomon*, *Fjellbergella*, *Nipponentomon*, *Tuxenentulus*, *Verrucoentomon* are the dominant taxa composing 66% of all species.

**Table 5.** List of Protura from RFE and their distributions.

Classification	Species	RFE	China	Korea	Japan
**Acerentomata** Yin, 1996					
**Berberentulidae** Yin, 1983					
***Baculentulus*** Tuxen,1977	*Baculentulus krabbensis* sp. n.*	5**			
	*Baculentulus loxoglenus* Yin, 1980	3, 4	+		
	*Baculentulus morikawai* (Imdadaté & Yosii, 1956)	3, 5, 7	+	+	+
	*Baculentulus pomorskii* Shrubovych, 2010*	4, 5, 6			
	*Baculentulus potapovi* Shrubovych, 2010*	4, 5, 6			
	*Baculentulus samchonri* Imdadaté & Szeptycki, 1976	5, 7		+	
**Acerentomidae** Silvestri, 1907					
***Acerentulus*** Berlese, 1908	*Acerentulus kisonis* Imdadaté, 1961	7			+
***Filientomon*** Rusek, 1974	*Filientomon duodecimsetosum* Nakamura, 2004*	3			
	*Filientomon gentaroanum* Nakamura, 2001	5			+
	*Filientomon takanawanum* (Imdadaté, 1956)	2, 4	+	+	+
***Fjellbergella*** Nosek, 1978	*Fjellbergella lazovskiensis* sp. n.*	7			
***Tuxenentulus*** Imdadaté, 1974	*Tuxenentulus ohbai* Imdadaté, 1974	2	+		+
	*Tuxenentulus solncevae* Shrubovych & Bernard, 2013*	8			
***Yamatentomon*** Imdadaté, 1964	*Yamatentomon kunnepchupi* Imadaté, 1964	3			+
	*Yamatentomon yamato* Imadaté & Yosii, 1956	3, 4, 5, 7	+	+	+
***Yichunentulus*** Yin, 1980	*Yichunentulus alpatovi* sp. n.*	7			
	*Yichunentulus borealis* (Nakamura, 2004), comb. n.*	3			
**Nipponentomidae** Yin, 1996					
***Callientomon*** Yin, 1980	*Callientomon chinensis* Yin, 1980	4, 5, 7	+		
***Imadateiella*** Rusek, 1974	*Imadateiella sharovi* (Martynova, 1977)*	1, 2			
***Nipponentomon*** Imdadaté & Yosii, 1959	*Nipponentomon bidentatum* Nakamura, 2004	3	+		
	*Nipponentomon* cf. *bidentatum* Nakamura, 2004*	2, 3			
	*Nipponentomon* cf. *heterothrixi* Yin & Xie, 1993*	5			
	*Nipponentomon khabarovskense* Nakamura, 2004*	2, 3, 7			
	*Nipponentomon jaceki* Shrubovych, 2009*	4			
	*Nipponentomon nippon* (Yoshii, 1938)	5, 6,7	+	+	+
***Verrucoentomon*** Rusek, 1974	*Verrucoentomon kawakatsui* Imadaté, 1964	3			+
	*Verrucoentomon louisanne* Shrubovych, 2012*	4			
	*Verrucoentomon shirampa* Imadaté, 1964	3, 4		+	+
**Eosentomata** Yin, 1996					
**Eosentomidae** Berlese, 1909					
***Eosentomon*** Berlese, 1909	*Eosentomon asahi* Imadaté, 1961	3, 4	+	+	+
	*Eosentomon brevicorpusculum* Yin, 1965	3, 7	+		
	*Eosentomon orientale* Yin, 1965	7	+		

* Known only from RFE so far.

** Numbers indicate the localities given in the materials and methods.

Compared with neighbouring regions, the Protura fauna of RFE is closely related to the fauna of Northeast China, Korea, and Japan, sharing 11, 7, and 11 species with each respectively (Bu et al. 2013, Imadaté 1974, Lee and Rim 1988, Szeptycki 2007, Yin 1999) (Table 5).

**Figure 9.**
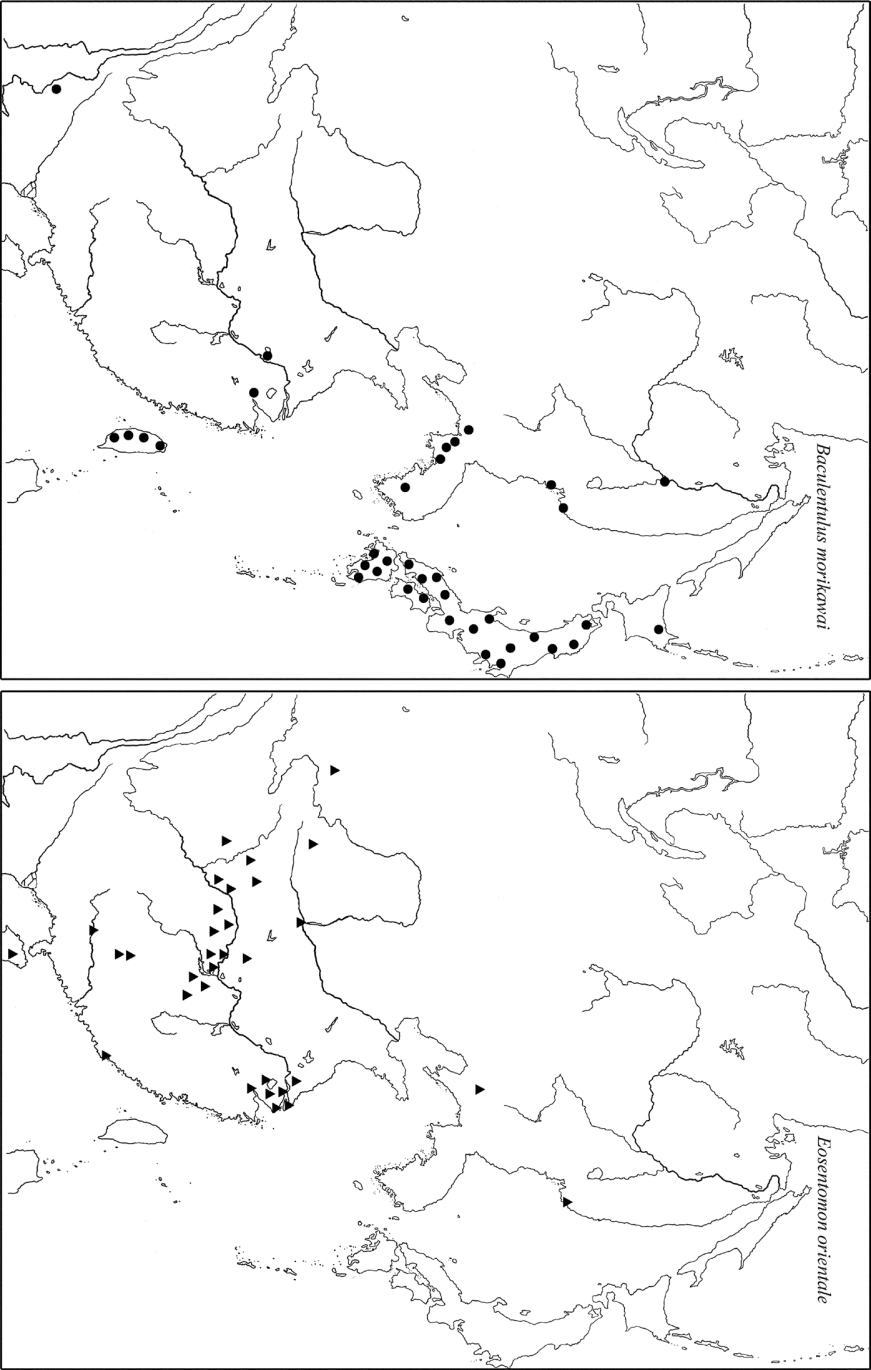
Distribution of two widely distributed East-Asiatic species of RFE. In addition to locations listed in the text, other records are used after Imadaté (1974), Lee and Rim (1988), Szeptycki (2007) and Yin (1999).

**Figure 10.**
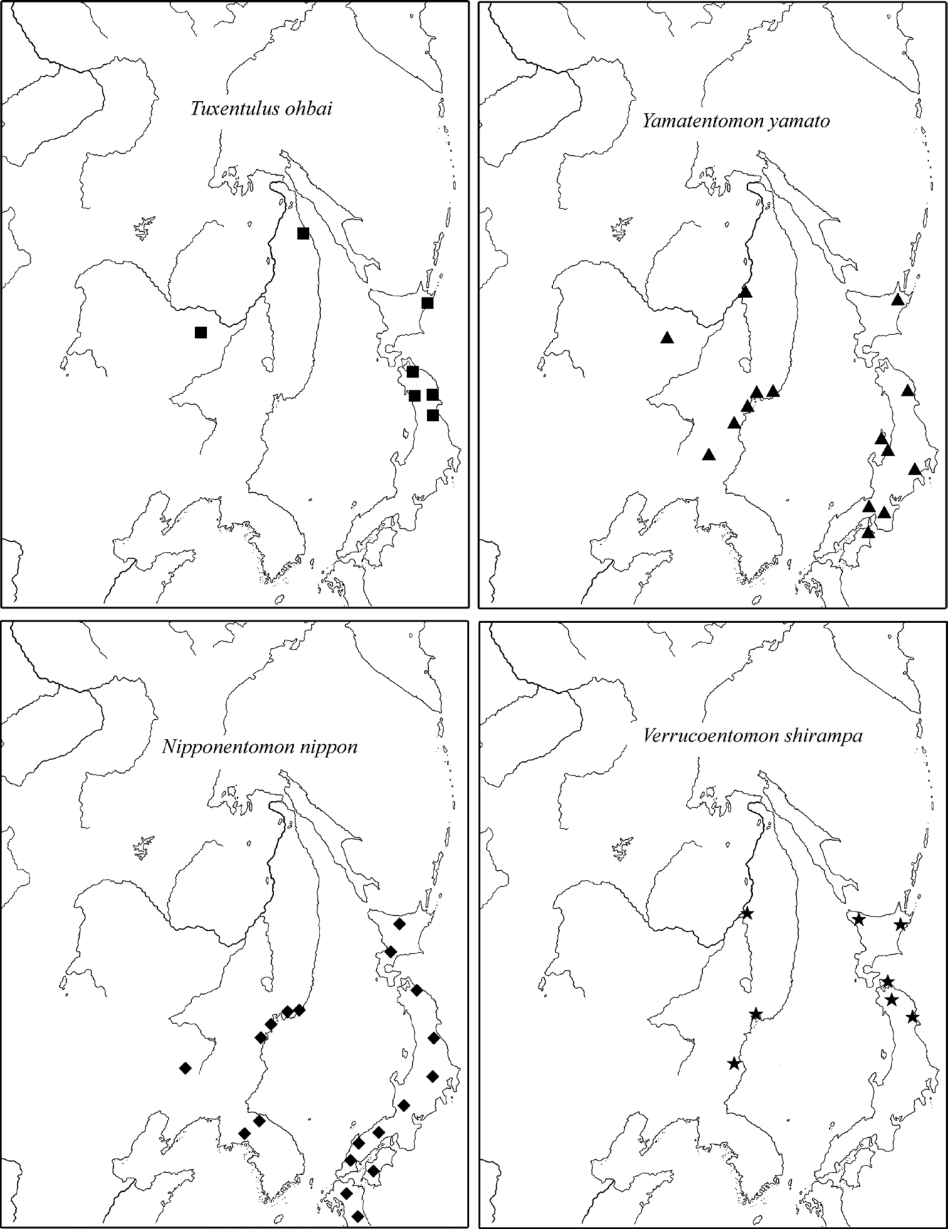
Distribution of typical East-Palearctic species of RFE. In addition to locations listed in the text, other records are used after Imadaté (1974), Lee and Rim (1988), Szeptycki (2007) and Yin (1999).

**Figure 11.**
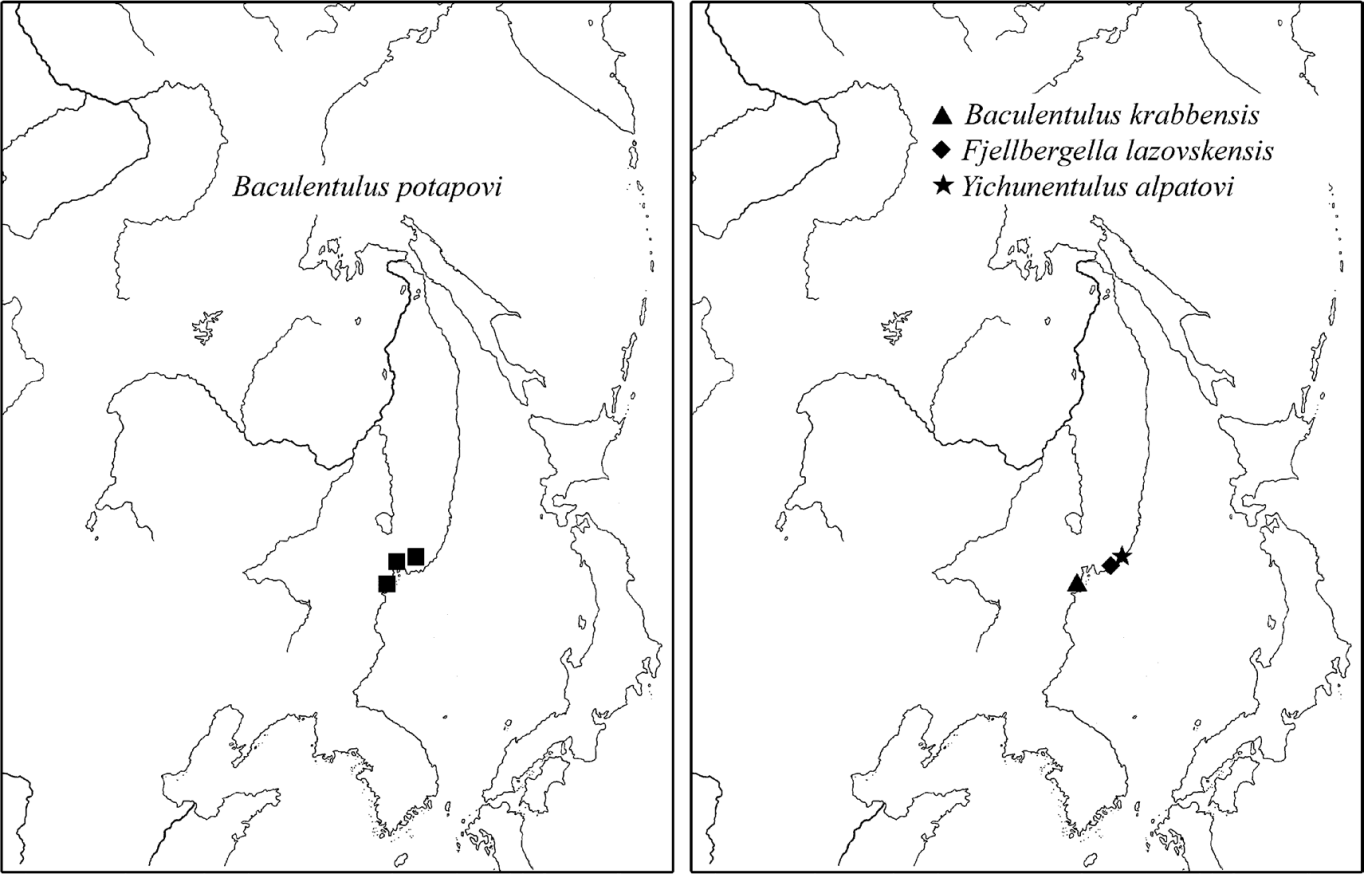
Distribution of some local species of RFE.

Based on the distributional data available so far, the species of Protura recorded in RFE can be classified into three groups:

1) Species widespread in the Eastern Asia including both its temperate and tropical parts (Fig. 9): *Baculentulus morikawai* (Imdadaté & Yosii, 1956), *Filientomon takanawanum* (Imdadaté, 1956), *Eosentomon brevicorpusculum* Yin, 1965 and *Eosentomon orientale* Yin, 1965.

2) Temperate East Palearctic species: *Baculentulus loxoglenus* Yin, 1980, *Baculentulus samchonri* Imdadaté & Szeptycki, 1976, *Acerentulus kisonis* Imdadaté, 1961, *Filientomon gentaroanum* Nakamura, 2001, *Tuxenentulus ohbai* Imdadaté, 1974, *Yamatentomon yamato* Imadaté & Yosii, 1956, *Yamatentomon kunnepchupi* Imadaté, 1964, *Callientomon chinensis* Yin, 1980, *Nipponentomon bidentatum* Nakamura, 2004, *Nipponentomon nippon* (Yoshii, 1938), *Verrucoentomon kawakatsui* Imadaté, 1964, *Verrucoentomon shirampa* Imadaté, 1964, and *Eosentomon asahi* Imadaté, 1961. The species are recorded in RFE, Northeast China, Korea and temperate part of Japan (Fig. 10).

3) Local species: *Baculentulus krabbensis* sp. n., *Baculentulus pomorskii* Shrubovych, 2010, *Baculentulus potapovi* Shrubovych, 2010, *Fjellbergella duodecimsetosum* Nakamura, 2004, *Fjellbergella lazovskiensis* sp. n., *Tuxenentulus solncevae* Shrubovych & Bernard, 2013, *Yichunentulus alpatovi* sp. n., *Yichunentulus borealis* (Nakamura, 2004), comb. n., *Imadateiella sharovi* (Martynova, 1977), *Nipponentomon* cf. *bidentatum* Nakamura, 2004, *Nipponentomon* cf. *heterothrixi* Yin & Xie, 1993, *Nipponentomon khabarovskense* Nakamura, 2004, *Nipponentomon jaceki* Shrubovych, 2009, *Verrucoentomon louisanne* Shrubovych & Bernard 2012 and *Eosentomon* sp. Nakamura, 2004. So far they have been recorded only in the south of RFE apart from *Imadateiella sharovi* distributed wider penetrating to the northern part of RFE (Fig. 11).

The biogeographical composition of Russian Far East, including Primorsky Krai and Khabarovsk Krai, has been described for many arthropods (Belyaev 2011, Kupyanskaya 2011, Loktionov 2011, Mutin 2011, Nemkov 2011, Proshchalykin 2011, Ryabinin 2009, Storozhenko 2011, Teslenko 2007, Wang et al. 2009). Strict detailed comparison of different taxa is not possible since the authors usually used different although similar chorological nomenclatures. Generalizing the published data mentioned above, the Primorsky Krai, the region in which the Protura was mostly studied by us, shows a high portion of species distributed only in eastern parts of Asia. These species are usually called as “far-eastern” (several subgroups can be involved to this group), manchurian etc. In fact, all species of Protura recorded in RFE belong to this “far-eastern” group in wide understanding although an exact biogeographical state of them can not be understood for now, especially in group of “local” species (see above). At a species level, the unexpected thing is that neither Holarctic nor Palearctic species of Protura is found by us. In invertebrates the Holarctic and trans-Palearctic groups of species take the considerable portion, from 10 to 55% (Table 6).

**Table 6.** The portion of Holarctic and trans-Palearctic species in fauna of arthropods of Primorsky Krai.

Taxa	Holarctic and trans-Palearctic species	References
Geometridae (Insecta: Lepidoptera)	~28%	Belyaev 2011
Formicidae (Insecta: Hymenoptera)	~14%	Kupyanskaya 2011
Pompilidae (Insecta: Hymenoptera)	~30%	Loktionov 2011
Syrphidae (Insecta: Diptera)	~37%	Mutin 2011
Spheciformes (Insecta: Hymenoptera)	~45%	Nemkov 2011
Apiformes (Insecta: Hymenoptera)	~43%	Proshchalykin 2011
Orthoptera (Insecta)	~14%	Storozhenko 2011
Plecoptera (Insecta)	~10%	Teslenko 2007
Oribatei (Arachnida)	~55%	Ryabinin 2009
Protura (Hexapoda)	0%	this study

Only three widely distributed species of Protura are known so far: *Acerentulus confinis* (Berlese, 1908), *Berberentulus capensis* (Womersley, 1931), and *Gracilentulus gracilis* (Berlese, 1908). These species are cosmopolitans in the broad sense, but their distribution is not fully understood (Szeptycki 2007). Two of them (*Acerentulus confinis* and *Gracilentulus gracilis*) are recorded from many European countries and, more rarely, from few other regions (Africa, North America, Australia, and New Zealand). *Berberentulus capensis* is scarcely recorded in warmer regions of the whole world. The reliable records of these species are unknown in Eastern Asia and, particularly, in RFE, but their presence is possible.

Several reasons can be proposed to explain the absence of the Holarctic and Palearctic species of Protura:

- High sensitivity of Protura to anthropogenic impact (Andrés 1999, Hågvar and Abrahamsen 1990, Niijima 1976, Parisi et al. 2005, Rusek 2007). The species preferring the disturbed sites are unknown in this group of animals. As a rule, in disturbed sites the portion of wider distributed and generalist species is greater while the endemic species decline, thus we can expect higher portion of endemic species in fauna of Protura. Strong negative effect of human practices on endemic species was shown, for instance, for the Collembola, the group of arthropods closely related to Protura (Cassagne et al. 2006, Deharveng 1996).

- In the Northern hemisphere, this group sharply declines at higher latitudes. In many groups of animals the portion of endemic species increases southwards in Eurasia (Chernov 1975) therefore we can assume higher endemism in Protura. The sharp decline from south to north along global transect of RFE is, however, shown for the most taxa of animals – for example, according to generalized analysis of Chernov et al. (2011) number of species of all insects in Chukotka (the most northern region) is about 20 times less than in Primorsky Krai (the most southern region).

- Low dispersal ability of Protura. This reason is possible but could not be substantiated since widely distributed species are known in other groups with low active movement but with high possibility of passive carriage (Coulson et al. 2002, Thibaud 2007). The passive carriage is unknown in Protura but only supposed by Yin et al. (1994).

In conclusion, the Protura appear to be a group with a low level of biogeographical “noise” (ruderal species), with high endemism and are candidate organisms for more detailed biogeographical analysis when more information is available for other regions.

## Supplementary Material

XML Treatment for Baculentulus krabbensis

XML Treatment for Fjellbergella lazovskiensis

XML Treatment for Yichunentulus alpatovi

XML Treatment for Acerentulus

XML Treatment for Fjellbergella

XML Treatment for Fjellbergella jilinensis

XML Treatment for Yichunentulus

XML Treatment for Yichunentulus borealis

XML Treatment for Imadateiella sharovi

XML Treatment for Baculentulus loxoglenus

XML Treatment for Baculentulus morikawai

XML Treatment for Baculentulus samchonri

XML Treatment for Baculentulus potapovi

XML Treatment for Acerentulus kisonis

XML Treatment for Filientomon gentaroanum

XML Treatment for Filientomon takanawanum

XML Treatment for Tuxenentulus ohbai

XML Treatment for Yamatentomon yamato

XML Treatment for Callientomon chinensis

XML Treatment for Verrucoentomon louisanne

XML Treatment for Verrucoentomon shirampa

XML Treatment for Nipponentomon cf. bidentatum

XML Treatment for Nipponentomon cf. heterothrixi

XML Treatment for Nipponentomon khabarovskense

XML Treatment for Nipponentomon jaceki

XML Treatment for Nipponentomon nippon

XML Treatment for Eosentomon asahi

XML Treatment for Eosentomon brevicorpusculum

XML Treatment for Eosentomon orientale
